# Trefoil Factor-3 (TFF3) Stimulates *De Novo* Angiogenesis in Mammary Carcinoma both Directly and Indirectly via IL-8/CXCR2

**DOI:** 10.1371/journal.pone.0141947

**Published:** 2015-11-11

**Authors:** Wai-Hoe Lau, Vijay Pandey, Xiangjun Kong, Xiao-Nan Wang, ZhengSheng Wu, Tao Zhu, Peter E Lobie

**Affiliations:** 1 Department of Pharmacology, Yong Loo Lin School of Medicine, National University of Singapore, Singapore, Singapore; 2 Cancer Science Institute of Singapore, National University of Singapore, Singapore, Singapore; 3 Hefei National Laboratory for Physical Sciences at Microscale and School of Life Sciences, University of Science and Technology of China, Hefei, Anhui, PR China; 4 Department of Pathology, Anhui Medical University, Hefei, Anhui, PR China; 5 Laboratory of Pathogenic Microbiology and Immunology, Anhui Medical University, Hefei, Anhui 230032, PR China; 6 National University Cancer Institute of Singapore, National Health System, National University of Singapore, Singapore, Singapore; University of Nebraska Medical Center, UNITED STATES

## Abstract

Mammary carcinoma cells produce pro-angiogenic factors to stimulate angiogenesis and tumor growth. Trefoil factor-3 (TFF3) is an oncogene secreted from mammary carcinoma cells and associated with poor prognosis. Herein, we demonstrate that TFF3 produced in mammary carcinoma cells functions as a promoter of tumor angiogenesis. Forced expression of TFF3 in mammary carcinoma cells promoted proliferation, survival, invasion and *in vitro* tubule formation of human umbilical vein endothelial cells (HUVEC). MCF7-TFF3 cells with forced expression of TFF3 generated tumors with enhanced microvessel density as compared to tumors formed by vector control cells. Depletion of TFF3 in mammary carcinoma cells by siRNA concordantly decreased the angiogenic behavior of HUVEC. Forced expression of TFF3 in mammary carcinoma cells stimulated IL-8 transcription and subsequently enhanced IL-8 expression in both mammary carcinoma cells and HUVEC. Depletion of IL-8 in mammary carcinoma cells with forced expression of TFF3, or antibody inhibition of IL-8, partially abrogated mammary carcinoma cell TFF3-stimulated HUVEC angiogenic behavior *in vitro*, as did inhibition of the IL-8 receptor, CXCR2. Depletion of STAT3 by siRNA in MCF-7 cells with forced expression of TFF3 partially diminished the angiogenic capability of TFF3 on stimulation of cellular processes of HUVEC. Exogenous recombinant hTFF3 also directly promoted the angiogenic behavior of HUVEC. Hence, TFF3 is a potent angiogenic factor and functions as a promoter of *de novo* angiogenesis in mammary carcinoma, which may co-coordinate with the growth promoting and metastatic actions of TFF3 in mammary carcinoma to enhance tumor progression.

## Introduction

Angiogenesis is required for expansion and metastatic progression of mammary carcinoma [1, 2]. Increased microvessel density and the presence of tumor metastases in lymph nodes predicts poor survival outcome in patients with mammary carcinoma [2–5]. Adequate vascularization of the tumor is required for provision of nutrients and oxygen to the growing tumor in a hypoxic microenvironment. Hypoxia results in production of pro-angiogenic factors that promote subsequent neovascularization [6, 7]. Establishment of highly permeable and disorganized vasculature within the tumor facilitates metastasis of cancer cells, which initially involves intravasation to adjacent vasculature [8, 9].

TFF3 is an estrogen regulated gene in mammary carcinoma and its TFF3 expression is generally positively associated with mammary carcinoma of the estrogen receptor positive (ER+) subtype [10, 11]. Increased TFF3 expression is observed in both non-invasive and invasive mammary carcinoma [12, 13]. Elevated TFF3 expression has also been reported in the molecular apocrine subtype of estrogen receptor negative (ER-) mammary carcinoma under androgen control [10, 14, 15]. Forced expression of TFF3 in mammary carcinoma cells has been demonstrated to promote oncogenicity, cellular invasion and resistance to apoptosis [12, 16, 17]. There is accumulated evidence that the expression of TFF3 is positively correlated with metastasis of mammary carcinoma [18, 19]. TFF3 expression in mammary carcinoma was reported to independently predict lymphovascular invasion and dissemination to lymph nodes [18]. Further, TFF3 expression is associated with poor survival outcome in patients with ER+ mammary carcinoma [19]. Functionally, TFF3 has recently been demonstrated to stimulate cellular invasion and metastasis of ER+ mammary carcinoma cells in a Src-STAT3 dependent manner [19]. TFF3 has been implicated as a pro-angiogenic factor due to promotion of capillary vessel formation in a chorioallantoic membrane (CAM) assay [20]. Furthermore, TFF3 expression is associated with increased microvessel density, both in gastric [21] and mammary carcinoma [18]. However, a functional role for TFF3 in tumor angiogenesis has not been determined.

IL-8 is a pro-angiogenic cytokine functionally involved in angiogenesis and metastasis of mammary carcinoma [22, 23]. It has been reported that elevated IL-8 expression in mammary carcinoma cells is significantly associated with angiogenesis and metastatic potential [24]. IL-8 is also associated with a higher tumor load, involvement of lymph node or liver and a worse outcome [25]. IL-8 has been demonstrated to promote the transcriptional activity of multiple genes involved in cell survival, migration, invasion and angiogenesis [26]. The functional role of TFF3 in *de novo* angiogenesis in mammary carcinoma has not yet been determined. Herein, we report that TFF3 secreted from mammary carcinoma cells promotes *de novo* angiogenesis, both directly by TFF3 stimulation of endothelial cells and indirectly via enhanced IL-8 expression, which subsequently regulates endothelial cell function.

## Results

### Forced expression of TFF3 in mammary carcinoma cells promoted angiogenic behavior of HUVEC

MCF-7 and T47D cells endogenously expressed moderate levels of TFF3 mRNA (Fig A in S1 File) as well as TFF3 protein present in both cell lysate and secreted to the media (Fig B in S1 File). In contrast, HUVEC endogenously expressed undetectable or very low level of TFF3 mRNA (Fig C in S1 File) and TFF3 protein (Fig D in S1 File) as compared with MCF-7 cells. To determine the effect of TFF3 secreted from mammary carcinoma cells on the angiogenic behavior of HUVEC, MCF-7 and T47D cells were stably transfected with a pIRESneo3 expression vector containing TFF3 cDNA or a pIRESneo3 empty vector [19]. The construct used to force the expression of TFF3 contains the canonical secretion sequence to direct secretion of TFF3 as previously described [16]. The expression of TFF3 in these new set of stable cell lines were again validated by semi-quantitative RT-PCR and Western blot analyses, which were consistent with our previous studies [16, 19]. Forced expression of TFF3 in MCF-7 cells increased TFF3 mRNA (Fig E in S1 File) and TFF3 protein (Fig F in S1 File) when compared with the control MCF7-Vec. Concordantly, forced expression of TFF3 in T47D cells increased TFF3 mRNA (Fig A in S2 File) and TFF3 protein when compared with the control T47D-Vec (Fig B in S2 File).

**Fig 1:**
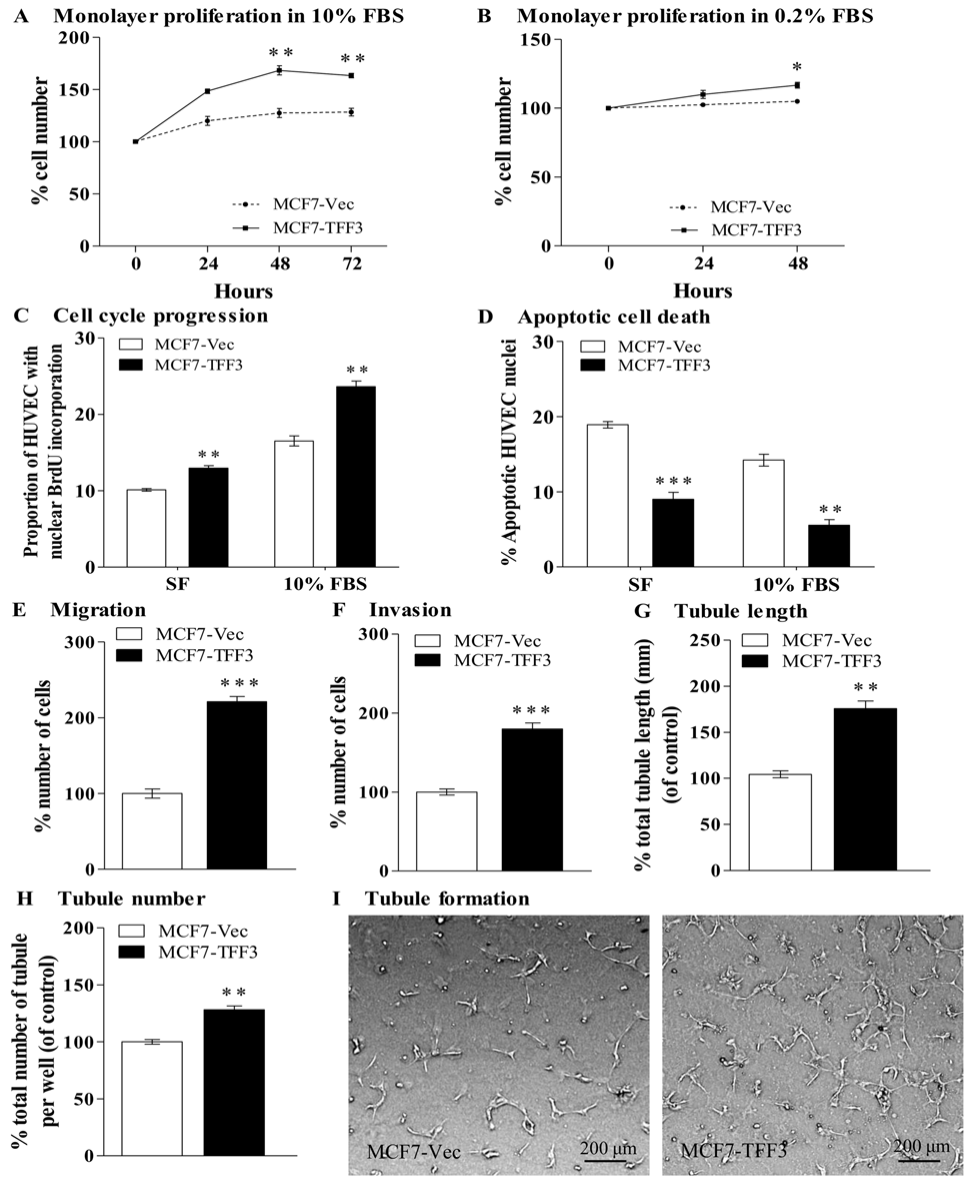
Mammary carcinoma cells with forced expression of TFF3 increased angiogenic behavior of HUVEC. (A) Monolayer proliferation of HUVEC after co-culture with MCF-7 cells with forced expression of TFF3 in 10% FBS conditions. (B) Monolayer proliferation of HUVEC after co-culture with MCF-7 cells with forced expression of TFF3 in 0.2% FBS conditions. (C) HUVEC cell cycle progression after 24 hours co-culture with MCF-7 cells with forced expression of TFF3 in serum-free (SF) and 10% FBS conditions. (D) HUVEC apoptotic cell death after 24 hours co-culture with MCF-7 cells with forced expression of TFF3 in serum-free and 10% FBS conditions. (E) HUVEC migration after 24 hours co-culture with MCF-7 cells with forced expression of TFF3 in serum-free conditions. (F) HUVEC invasion after 24 hours co-culture with MCF-7 cells with forced expression of TFF3 in serum-free conditions. (G) and (H) HUVEC tubule formation *in vitro* in the Matrigel after 12 hours co-culture with MCF-7 cells with forced expression of TFF3. Total tubule length (G) and total tubule number (H) were assessed. (I) Representative light photomicrographs of HUVEC tubule formation *in vitro* in the Matrigel after 12 hours co-culture with MCF-7 cells with forced expression of TFF3. MCF-7 cells with empty vector (MCF7-Vec) was used as control. β-ACTIN was used as input control in semi-quantitative RT-PCR and Western blot analyses. *, *P < 0*.*05*; **, *P* < *0*.*01*; ***, *P* < *0*.*001*; scale bar, 200 μm.

Forced expression of TFF3 in mammary carcinoma cells has previously been demonstrated to promote proliferation, survival and invasion [16]. We further determined if TFF3 secreted from mammary carcinoma cells modulates the angiogenic behavior of HUVEC. By use of an indirect co-culture transwell system, MCF-7 cells with forced expression of TFF3 increased HUVEC monolayer proliferation in 10% FBS conditions by 35% when compared with the control MCF7-Vec (Fig 1A). MCF-7 cells with forced expression of TFF3 induced a small but significantly increased HUVEC monolayer proliferation in 0.2% FBS conditions by 12% when compared with the control MCF7-Vec (Fig 1B). Increased monolayer cell proliferation is achieved by increased cell cycle progression and/or decreased apoptotic cell death. Cell cycle progression was analyzed by 5-bromo-2-deoxyuridine (BrdU) incorporation. Forced expression of TFF3 in MCF-7 cells increased HUVEC cell cycle progression, in both serum-free and 10% FBS conditions by 28% and 43%, respectively when compared with the control MCF7-Vec (Fig 1C and Fig G in S1 File). Apoptotic cell death was analyzed by fluorescent microscopic analysis of nuclear staining patterns with Hoechst 33258 dye. Forced expression of TFF3 in MCF-7 cells significantly decreased HUVEC apoptotic cell death, in both serum-free and 10% FBS conditions by 52% and 61%, respectively when compared with control MCF7-Vec cells (Fig 1D and Fig H in S1 File). Hence, TFF3 secreted from mammary carcinoma cells promoted HUVEC cell proliferation and survival.

Forced expression of TFF3 in MCF-7 cells significantly increased HUVEC migration and invasion by 121% and 80%, respectively as compared with the control MCF7-Vec (Fig 1E and 1F). HUVEC retain the ability to form three-dimensional tubules in the matrix-rich basement membrane extract Matrigel and is indicative of angiogenesis *in vitro* [27, 28]. We therefore determined if MCF-7 cells with forced expression of TFF3 stimulates tubule formation. Forced expression of TFF3 in MCF-7 cells significantly increased HUVEC tubule length and tubule number by 72% and 28%, respectively when compared with the control MCF7-Vec cells (Fig 1G–1I). Concordantly, T47D cells with forced expression of TFF3 significantly increased HUVEC monolayer cell proliferation, cell cycle progression, survival, migration, invasion and tubule formation *in vitro* (Fig C-K in S2 File).

### Forced expression of TFF3 in mammary carcinoma cells promoted angiogenesis *in vivo*

**Fig 2:**
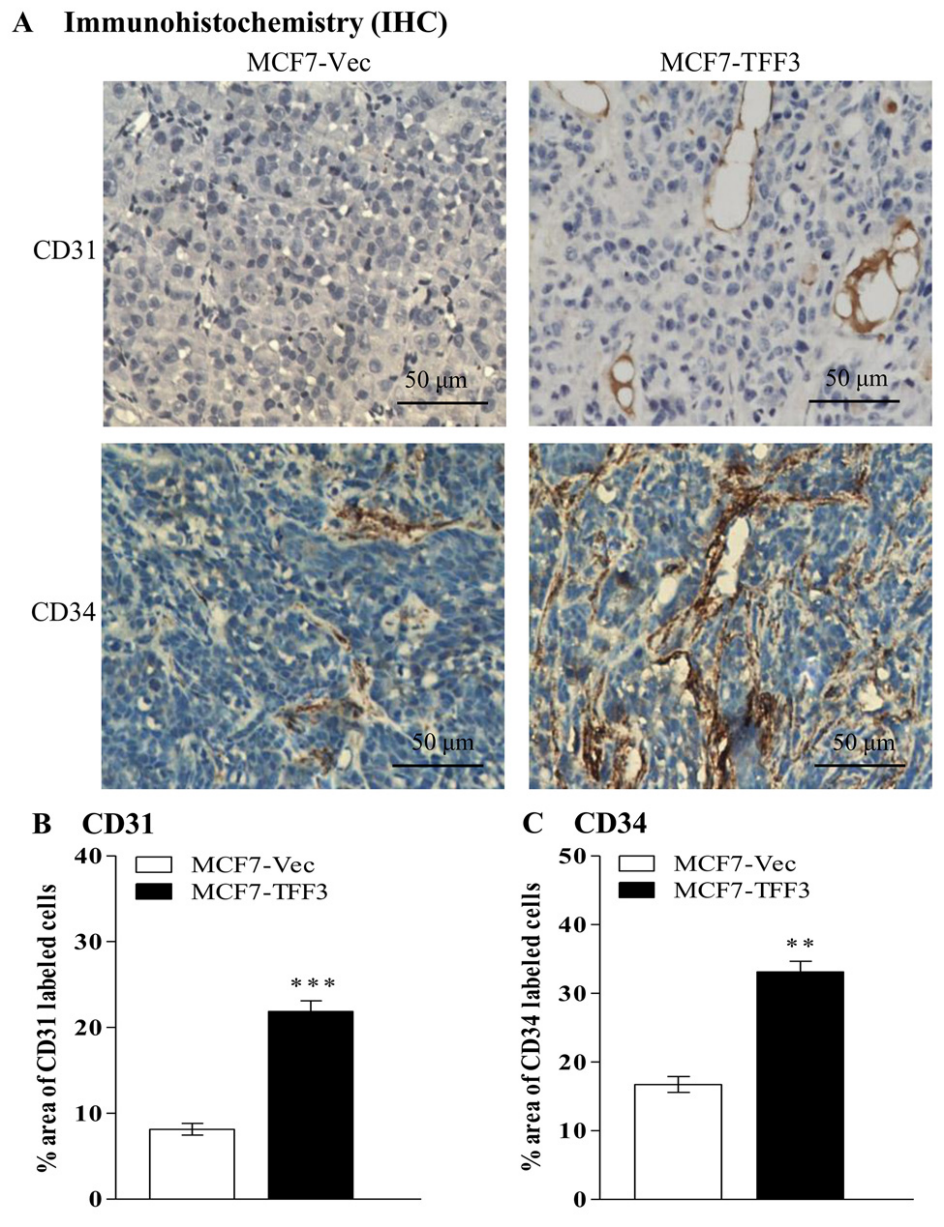
Mammary carcinoma cells with forced expression of TFF3 promoted tumor angiogenesis *in vivo*. (A) IHC analysis of CD31 and CD34 protein expressions in xenograft tumors formed by MCF-7 cells with forced expression of TFF3. (B) Microvessel density (CD31) was assessed by quantifying percentage area of CD31 labeled cells in xenografts formed by MCF-7 cells with forced expression of TFF3. (C) CD34 was assessed by quantifying percentage area of CD34 labeled cells in xenografts formed by MCF-7 cells with forced expression of TFF3. MCF-7 cells with empty vector (MCF7-Vec) used as control. **, *P* < *0*.*01*; ***, *P* < *0*.*001*; scale bar, 50 μm.

Increased TFF3 expression in MCF-7 cells has been demonstrated to enhance tumor growth in xenograft models [16]. The expression of CD31 and CD34 in tumors formed by MCF-7 cells with forced expression of TFF3 and control vector cells were analyzed by Immunohistochemistry (IHC). Microvessel density in xenografts may be quantified by the area of CD31 and/or CD34 labeled cells. CD31 is an endothelial cell surface marker and an indicator of angiogenesis [29] whereas CD34 (podoplanin) is a small transmembrane protein of lymphatic endothelium and a molecular marker of lymphangiogenesis [30]. MCF-7 cells with forced expression of TFF3 generated tumors with increased CD31 and CD34 labeled cells as compared to tumors formed by control cells (Fig 2A–2C). Hence, forced expression of TFF3 in MCF-7 cells increased tumor microvessel density and subsequently promoted angiogenesis *in vivo*.

### Depletion of TFF3 in mammary carcinoma cells decreased angiogenic behavior of HUVEC

**Fig 3:**
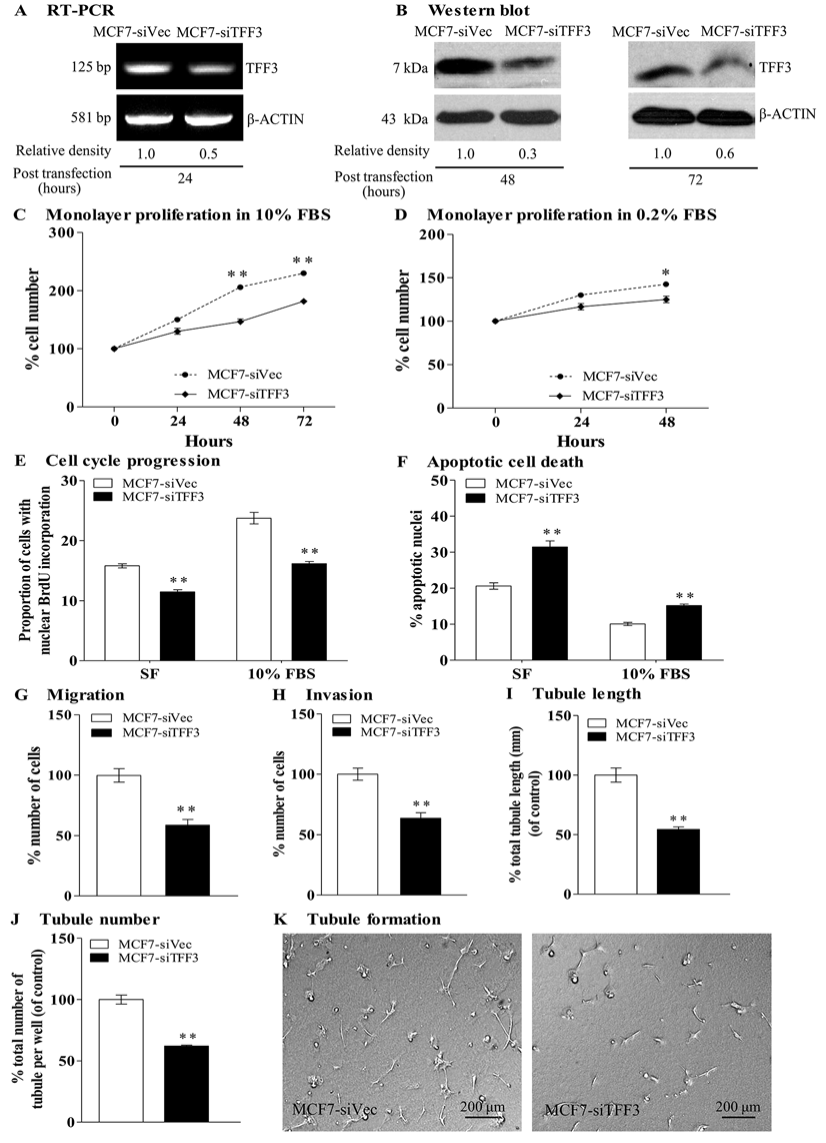
Depletion of TFF3 by siRNA in mammary carcinoma cells decreased angiogenic behavior of HUVEC. **(**A) Semi-quantitative RT-PCR analysis of TFF3 mRNA level in MCF-7 cells with depletion of TFF3 (MCF7-siTFF3) and control siRNA vector cells (MCF7-siVec) after 24 hours transient transfection. (B) Western blot analysis of TFF3 protein in MCF-7 cells with depletion of TFF3 and control siRNA vector cells after 48 and 72 hours transient transfection. (C) Monolayer proliferation of HUVEC after co-culture with MCF-7 cells with depletion of TFF3 in 10% FBS conditions. (D) Monolayer proliferation of HUVEC after co-culture with MCF-7 cells with depletion of TFF3 in 0.2% FBS conditions. (E) HUVEC cell cycle progression after 24 hours co-culture with MCF-7 cells with depletion of TFF3 in serum-free and 10% FBS conditions. (F) HUVEC apoptotic cell death after 24 hours co-culture with MCF-7 cells with depletion of TFF3 in serum-free and 10% FBS conditions. (G) HUVEC migration after 24 hours co-culture with MCF-7 cells with depletion of TFF3 in serum free conditions. (H) HUVEC invasion after 24 hours co-culture with MCF-7 cells with depletion of TFF3 in serum free conditions. (I) and (J) HUVEC tubule formation *in vitro* in the Matrigel after 12 hours co-culture with MCF-7 cells with depletion of TFF3 in serum-free conditions. Total tubule length (I) and tubule number (J) were assessed after 12 hours incubation. (K) Representative light photomicrographs of HUVEC tubule formation *in vitro* in the Matrigel after 12 hours co-culture with MCF-7 cells with depletion of TFF3. MCF-7 cells with control siRNA vector (MCF7-siVec) was used as control. β-ACTIN was used as input control in semi-quantitative RT-PCR and Western blot analyses. *, *P < 0*.*05*; **, *P* < *0*.*01*; ***, *P* < *0*.*001*; scale bar, 200 μm.

We next examined the effects of depletion of endogenous TFF3 in mammary carcinoma cells on the angiogenic behavior of HUVEC by use of siRNA. MCF-7 and T47D cells were transiently transfected with a pSilencer 2.1-U6 hygro expression vector containing TFF3 siRNA or with a pSilencer 2.1-U6 hygro empty vector containing control siRNA. Depletion of TFF3 in MCF-7 cells decreased TFF3 mRNA as compared with the control MCF7-siVec (Fig 3A). Similarly, MCF-7 cells with siRNA mediated depletion of TFF3 expressed lower level of TFF3 protein 48 and 72 hours after transient transfection, when compared with the control MCF7-siVec (Fig 3B). Concordantly, depletion of TFF3 by siRNA in T47D cells decreased TFF3 mRNA (Fig A in S3 File) and TFF3 protein (Fig B in S3 File) when compared with the control T47D-siVec.

Depletion of TFF3 in MCF-7 cells significantly decreased HUVEC monolayer proliferation, in both 10% FBS and 0.2% FBS conditions by 48% and 18%, respectively when compared with the control MCF7-siVec co-cultured with HUVEC (Fig 3C and 3D). Depletion of TFF3 in MCF-7 cells significantly decreased HUVEC cell cycle progression, in both serum-free and 10% FBS conditions by 27% and 32%, respectively when compared with the control MCF7-siVec (Fig 3E). Depletion of TFF3 in MCF-7 cells significantly increased HUVEC apoptotic cell death, in both serum-free and 10% FBS conditions by 53% and 50%, respectively when compared with the control MCF7-siVec (Fig 3F). Furthermore, depletion of TFF3 in MCF-7 cells significantly decreased HUVEC migration and invasion by 41% and 36%, respectively as compared to the control MCF7-siVec (Fig 3G and 3H). Finally, depletion of TFF3 in MCF-7 cells significantly decreased tubule length and tubule number by 46% and 38%, respectively when compared with the control MCF7-siVec (Fig 3I–3K). Concordantly, depletion of TFF3 in T47D cells also significantly decreased HUVEC monolayer cell proliferation, cell cycle progression, survival, migration, invasion, and tubule formation *in vitro* (Fig C-K in S3 File).

### Forced expression of TFF3 in mammary carcinoma cells enhanced IL-8 expression

**Fig 4:**
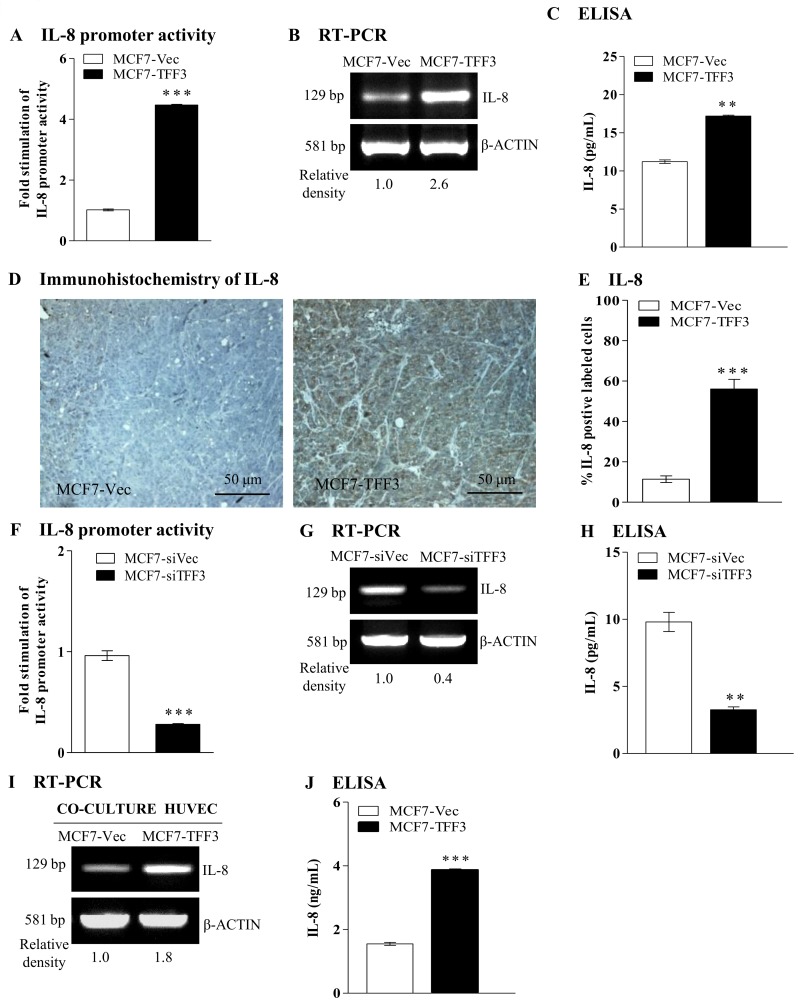
TFF3 enhanced IL-8 expression in mammary carcinoma cells and HUVEC. (A) IL-8 promoter reporter activity (full length, -4800 to + 104 bp) in MCF-7 cells with forced expression of TFF3 and control vector cells. MCF-7 cells with empty vector (MCF7-Vec) was used as control. (B) Semi-quantitative RT-PCR analysis of IL-8 mRNA level in MCF-7 with forced expression of TFF3 and control vector cells. (C) ELISA analysis of IL-8 protein secreted to the medium by MCF-7 cells with forced expression of TFF3 and control vector cells. (D) IHC analysis of IL-8 protein expression in xenograft tumors formed by MCF-7 cells with forced expression of TFF3. (E) Percentage of IL-8 labeled cells in xenograft tumors formed by MCF-7 cells with forced expression of TFF3 and control vector cells. (F) IL-8 promoter reporter activity (full length, -4800 to + 104 bp) in MCF-7 cells with depletion of TFF3 and control siRNA vector cells. MCF-7 cells with control siRNA vector (MCF7-siVec) was used as control. (G) Semi-quantitative RT-PCR analysis of IL-8 mRNA level in MCF-7 with depletion of TFF3 and control siRNA vector cells. (H) ELISA analysis of IL-8 protein secreted to the medium by MCF-7 cells with depletion of TFF3 and control siRNA vector cells. (I) Semi-quantitative RT-PCR analysis of IL-8 mRNA level in HUVEC co-cultured with MCF-7 cells with forced expression of TFF3 and control vector cells. (J) ELISA analysis of IL-8 protein secreted to the medium by HUVEC co-cultured MCF-7 with forced expression of TFF3 and control vector cells. β-ACTIN was used as input control in semi-quantitative RT-PCR and Western blot analyses. **, *P* < *0*.*01*; ***, *P* < *0*.*001*; scale bar, 50 μm.

According to the previously published microarray data set (Gene Expression Omnibus accession number GSE3494) [31] as well as the gene expression profiling of MCF-7 cells with forced expression of TFF3 [16], several angiogenic associated with tumor angiogenesis were selected for validation by real-time qPCR analysis. As observed in Fig A in S4 File, MCF-7 cells with forced expression of TFF3 increased gene expression of IL-8 (5.5-fold) as compared with the control MCF7-Vec. We next determined if TFF3 stimulates IL-8 promoter activity. Mammary carcinoma cells with forced expression of TFF3 were transiently transfected with an IL-8 promoter reporter (full length, -4800 to +104 bp) and a pRL-CMV control reporter vector. Forced expression of TFF3 in MCF-7 cells significantly increased IL-8 promoter activity (4.5-fold increase) when compared with the control MCF7-Vec (Fig 4A). Forced expression of TFF3 in MCF-7 cells increased IL-8 mRNA (Fig 4B) and secretion of IL-8 protein (Fig 4C) as compared with the control MCF7-Vec. In xenografts, MCF-7 cells with forced expression of TFF3 generated tumors with increased the expression of IL-8 protein as compared to tumors produced by control cells (Fig 4D). Quantitatively, MCF7-TFF3 cells generated tumors with increased IL-8 labeled cells (45%) as compared to the tumors formed by control MCF7-Vec (Fig 4E), indicative that TFF3 also enhanced IL-8 protein expression *in vivo*. Concordantly, T47D cells with forced expression of TFF3 increased IL-8 promoter activity (Fig B in S4 File) and subsequently enhanced the expression of IL-8 mRNA (Fig C in S4 File) and secretion of IL-8 protein (Fig D in S4 File). Depletion of TFF3 in MCF-7 cells significantly decreased IL-8 promoter activity (3.4-fold decrease) as compared to the control MCF7-Vec (Fig 4F). Furthermore, depletion of TFF3 in MCF-7 cells decreased the expression of IL-8 mRNA (Fig 4G) and secretion of IL-8 protein (Fig 4H) when compared with the control MCF7-siVec. Concordantly, depletion of TFF3 expression in T47D cells reduced IL-8 promoter activity (Fig E in S4 File) and subsequently decreased the expression of IL-8 mRNA (Fig F in S4 File) and secretion of IL-8 protein (Fig G in S4 File). Additionally, HUVEC co-cultured with MCF-7 cells with forced expression of TFF3 increased IL-8 mRNA (Fig 4I) and secretion of IL-8 protein (Fig 4J) as compared with HUVEC co-cultured with control MCF7-Vec. Therefore, TFF3 produced by mammary carcinoma cells enhances IL-8 expression in mammary carcinoma and endothelial cells.

### Depletion of mammary carcinoma cells IL-8 by siRNA abrogated the stimulatory effect of TFF3 on angiogenic behavior of HUVEC

**Fig 5:**
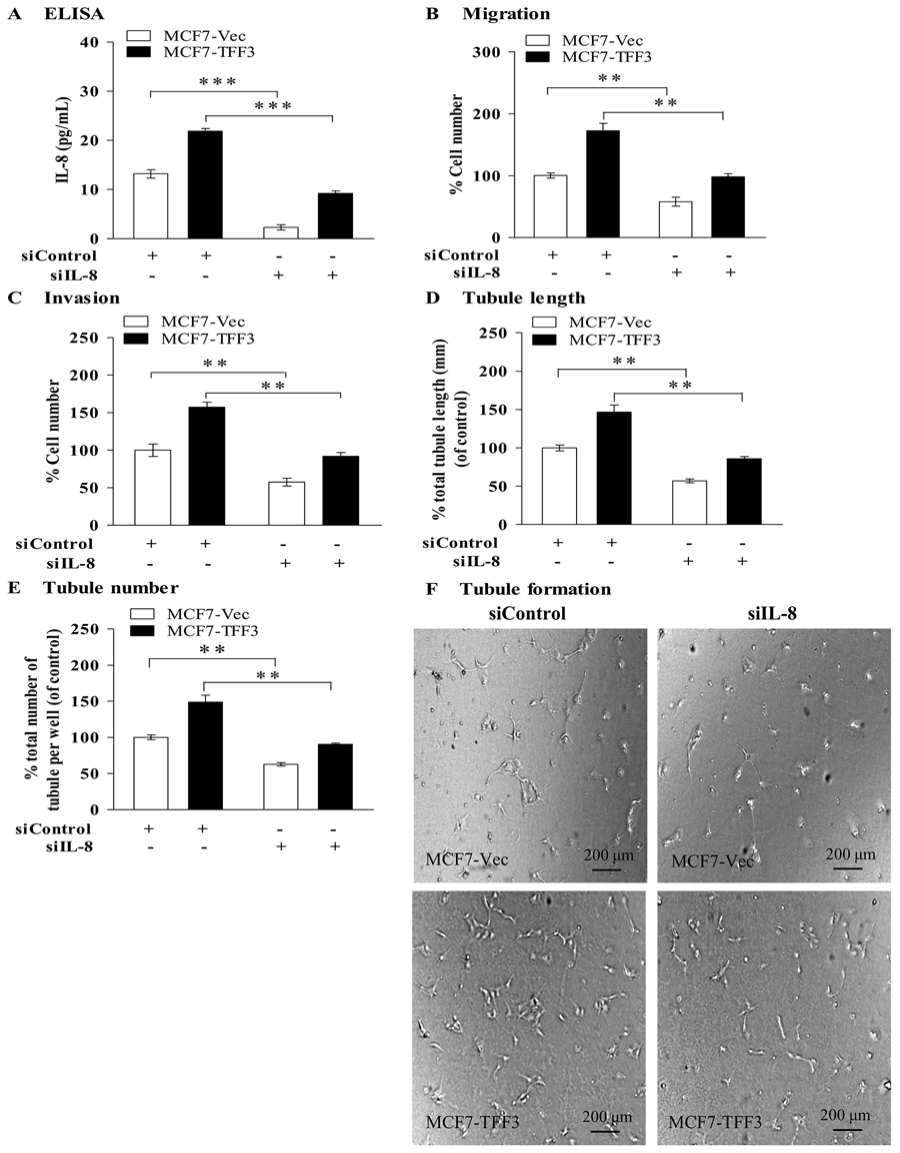
Depletion of IL-8 in mammary carcinoma cells with forced expression of TFF3 abrogated the stimulatory effect of TFF3 on HUVEC migration, invasion, and tubule formation *in vitro*. (A) ELISA analysis of IL-8 protein secreted to the medium by MCF7-Vec and MCF7-TFF3 transiently transfected with control siRNA and IL-8 siRNA after 48 hours transfection. Scrambled control siRNA (siControl) was used as control. MCF7-Vec transiently transfected with scrambled control siRNA was a baseline. (B) HUVEC migration after 24 hours co-culture with MCF-7 cells with forced expression of TFF3 transiently transfected with control siRNA and IL-8 siRNA in serum-free conditions. (C) HUVEC invasion after 24 hours co-culture with MCF-7 cells with forced expression of TFF3 transiently transfected with control siRNA and IL-8 siRNA in serum-free conditions. (D) and (E) HUVEC tubule formation *in vitro* in the Matrigel after 12 hours co-culture with MCF-7 cells with forced expression of TFF3 transiently transfected with control siRNA and IL-8 siRNA in serum-free conditions. Total tubule length (D) and tubule number (E) was assessed using ImageJ analysis software. (F) Representative light photomicrographs of HUVEC tubule formation *in vitro* in the Matrigel after 12 hours co-culture of with MCF-7 cells with forced expression of TFF3 transiently transfected with control siRNA and IL-8 siRNA. **, *P* < *0*.*01* as compared with MCF7-Vec or MCF7-TFF3 transiently transfected with scrambled control siRNA, respectively. Scale bar, 200 μm.

To determine if IL-8 mediates the stimulatory effect of TFF3 on the angiogenic behavior of HUVEC, an IL-8 siRNA was utilized to selectively deplete the expression of IL-8 in MCF-7 cells with forced expression of TFF3. IL-8 siRNA decreased the secretion of IL-8 protein from the cells (Fig 5A) when compared with the siControl cells. Depletion of IL-8 in MCF7-Vec significantly decreased HUVEC migration by 42% (Fig 5B) and invasion by 42% (Fig 5C) when compared with the MCF7-Vec transiently transfected with control siRNA, indicating that depletion of IL-8 by siRNA abrogated HUVEC migration and invasion. Depletion of IL-8 in MCF7-TFF3 significantly decreased HUVEC migration by 74% (Fig 5B) and invasion by 65% (Fig 5C) when compared with MCF7-TFF3 transiently transfected with control siRNA, indicating depletion of IL-8 by siRNA abrogated TFF3-stimulated HUVEC migration and invasion. Depletion of IL-8 in MCF7-Vec significantly decreased tubule length by 43% (Fig 5D) and tubule number by 37% (Fig 5E) when compared with MCF7-Vec transiently transfected with control siRNA and in MCF7-TFF3 significantly decreased tubule length by 61% and tubule number by 58% (Fig 5D–5F) when compared with MCF7-TFF3 transiently transfected with control siRNA.

### Inhibition of IL-8 with anti-IL-8 monoclonal antibody attenuated the stimulatory effect of TFF3 on angiogenic behavior of HUVEC

**Fig 6:**
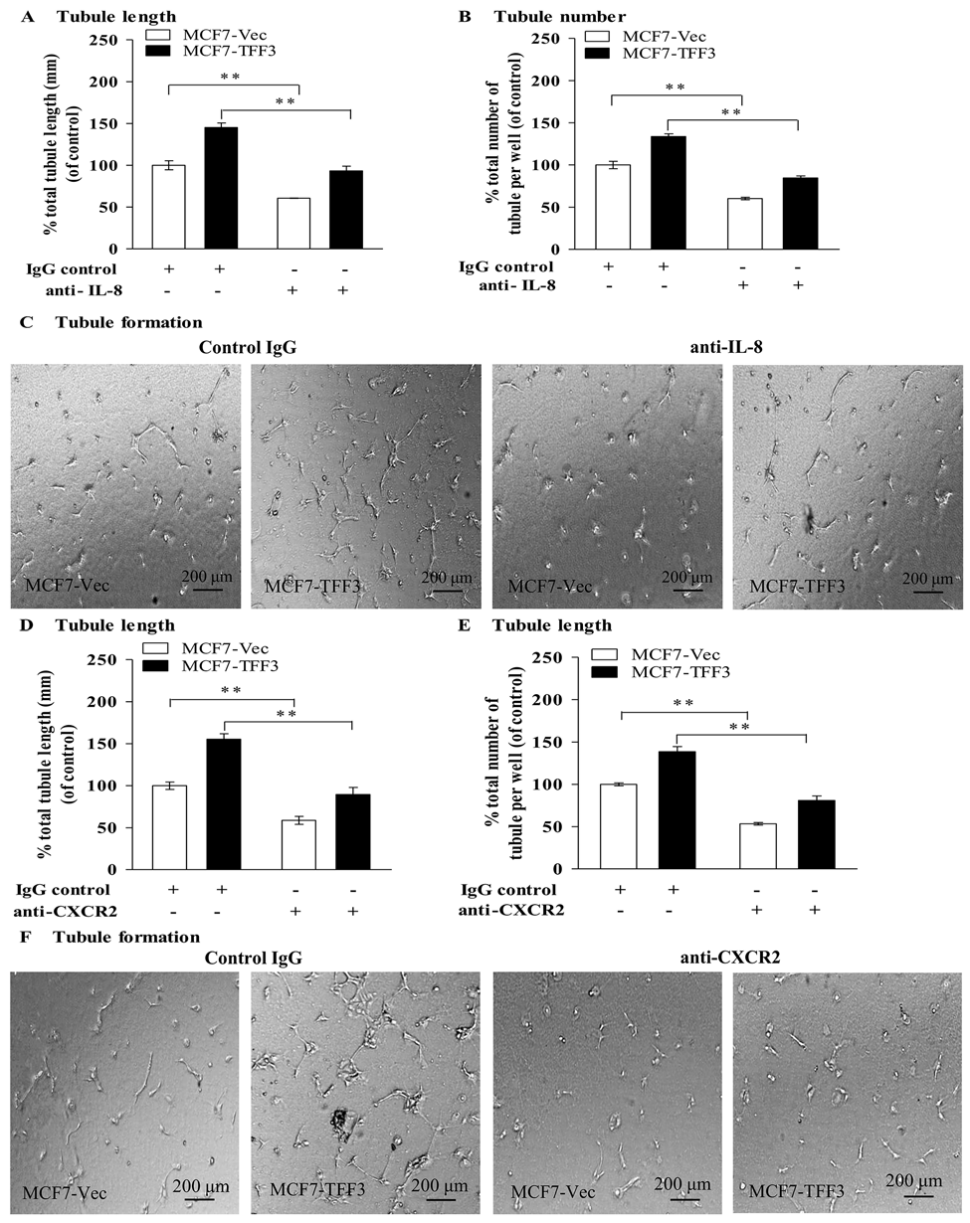
Monoclonal antibody inhibition of IL-8 and CXCR2 decreased TFF3 stimulated HUVEC tubule formation *in vitro*. (A) and (B), HUVEC tubule formation *in vitro* in the Matrigel after 12 hours co-culture with MCF-7 cells with forced expression of TFF3 treated with IgG control or 50 μg/mL of anti-IL-8 monoclonal antibody in serum-free conditions. IgG was used as control. HUVEC co-cultured with MCF7-Vec treated with IgG control was a baseline. Total tubule length (A) and tubule number (B) was assessed using ImageJ analysis software. (C) Representative light photomicrographs of HUVEC tubule formation *in vitro* in the Matrigel, in which HUVEC co-cultured with MCF7-Vec and MCF7-TFF3 treated with IgG control or 50 μg/mL of anti-IL-8 monoclonal antibody. **, *P* < *0*.*01* as compared to HUVEC co-cultured with MCF7-Vec or MCF7-TFF3 treated with IgG control. (D) and (E) HUVEC tubule formation *in vitro*, in which MCF-7 cells with forced expression of TFF3 co-cultured with HUVEC treated with IgG control or 20 μg/mL of anti-CXCR2 monoclonal antibody. MCF7-Vec co-cultured with HUVEC treated with IgG control was as baseline. Total tubule length (D) and tubule number (E) was assessed. (F) Representative light photomicrographs of HUVEC tubule formation *in vitro* in the Matrigel, in which MCF7-Vec and MCF7-TFF3 co-cultured with HUVEC treated with IgG control or 20 μg/mL of anti-CXCR2 monoclonal antibody. **, *P* < *0*.*01* as compared to MCF7-Vec or MCF7-TFF3 co-cultured with HUVEC treated with IgG control. Scale bar, 200 μm.

Humanized anti-IL-8 monoclonal antibodies (e.g., ABX-IL8) bind to IL-8 and inhibits interaction with and activation of receptors for IL-8 [32, 33]. Accordingly, an anti-IL-8 monoclonal antibody was utilized for inhibition of IL-8 in MCF-7 cells with forced expression of TFF3. HUVEC co-cultured with MCF7-Vec treated with the monoclonal antibody to IL-8 exhibited significantly decreased tubule length in a concentration-dependent manner, with 57% reduction of HUVEC tubule formation *in vitro* at 50 μg/mL of IL-8 monoclonal antibody (Fig A in S5 File). To determine if inhibition of IL-8 with IL-8 monoclonal antibody attenuates the stimulatory effect of TFF3 on HUVEC tubule formation *in vitro*, HUVEC were co-cultured with MCF7-Vec or MCF7-TFF3 treated with 50 μg/mL of IL-8 monoclonal antibody and IgG control (Fig 6A–6C). Inhibition of IL-8 in MCF7-Vec with IL-8 monoclonal antibody significantly decreased tubule length by 39% (Fig 6A) and tubule number by 40% (Fig 6B) when compared with MCF7-Vec treated with IgG control. Inhibition of IL-8 in MCF7-TFF3 with IL-8 monoclonal antibody significantly decreased tubule length by 52% (Fig 6A) and tubule number by 49% (Fig 6B) when compared with MCF7-TFF3 treated with IgG control.

### Blocking of CXCR2 in HUVEC with a CXCR2 monoclonal antibody inhibited IL-8 mediated angiogenic behavior of HUVEC stimulated by TFF3

CXCR1 and CXCR2, receptors for IL-8, are highly expressed in tumor and endothelial cells and potentially mediate the angiogenic function of IL-8 in tumor angiogenesis [34–36]. To determine if IL-8 mediated the effect of TFF3 on HUVEC tubule formation *in vitro* through either the CXCR1 or CXCR2 receptor, MCF7-Vec and MCF7-TFF3 were co-cultured with HUVEC treated with 20 μg/mL anti-CXCR2 (Fig 6D–6F) or anti-CXCR1 monoclonal antibody (Fig B-D in S5 File), respectively. Co-culture of MCF7-Vec with HUVEC treated with anti-CXCR2 monoclonal antibody decreased tubule length by 41% (Fig 6D) and tubule number by 47% (Fig 6E), when compared with HUVEC treated with IgG control. Co-culture of MCF7-TFF3 with HUVEC treated with anti-CXCR2 monoclonal antibody significantly decreased tubule length by 66% (Fig 6D) and tubule number by 58% (Fig 6E), when compared with HUVEC treated with IgG control. In contrast, HUVEC treated anti-CXCR1 monoclonal antibody, when co-cultured with MCF7-Vec or MCF7-TFF3, did not exhibit significant changes in tubule length or tubule number when compared with the respective cells co-cultured with HUVEC treated with IgG control (Fig B-D in S5 File).

### Depletion of STAT3 in mammary carcinoma cells by siRNA partially abrogated TFF3 stimulated angiogenic behavior of HUVEC

**Fig 7:**
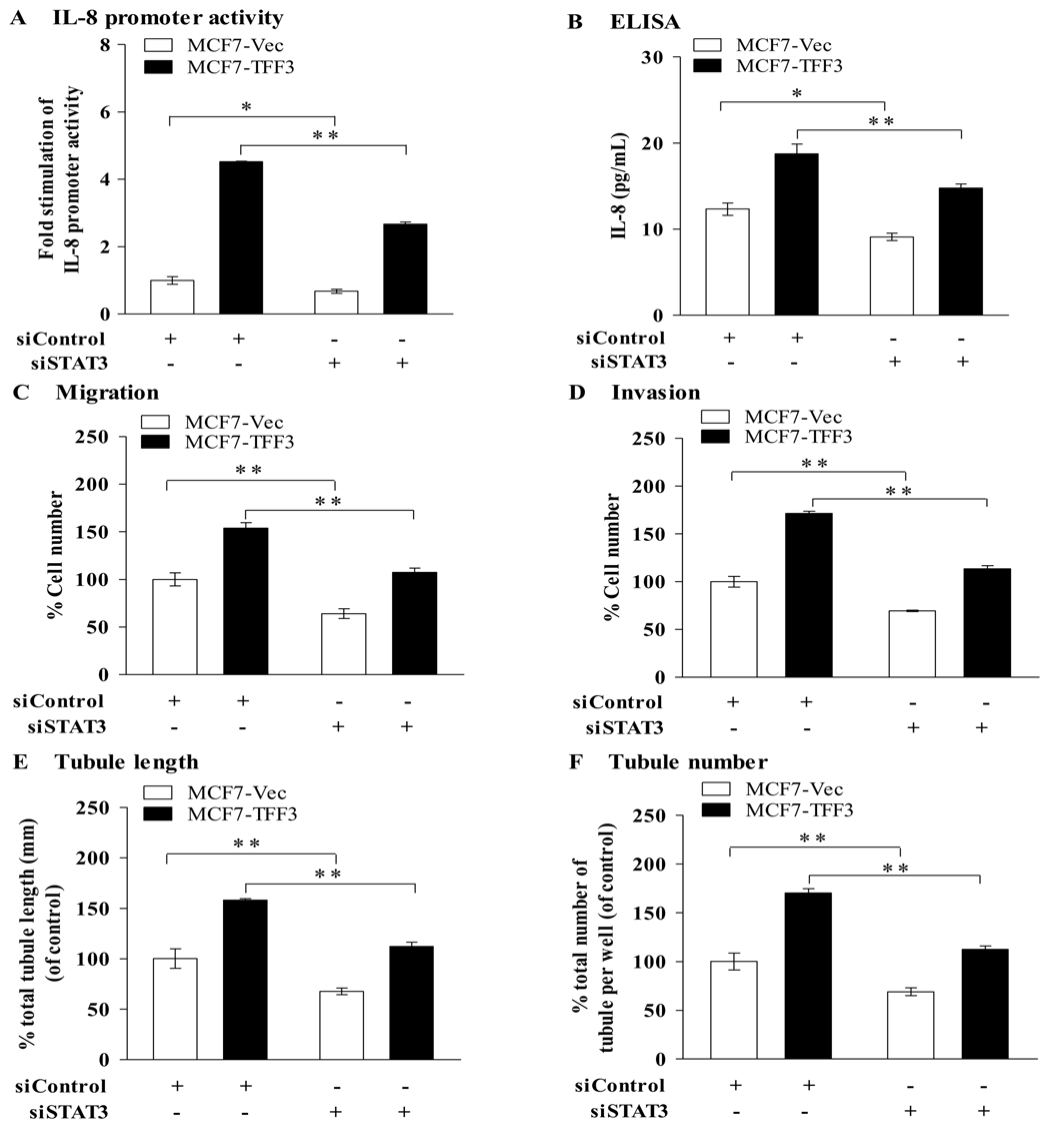
Depletion of STAT3 in mammary carcinoma cells by siRNA partially diminished the ability of TFF3 to stimulate mammary carcinoma cell IL-8 promoter activity and IL-8 protein expression as well as HUVEC migration, invasion, and tubule formation *in vitro*. (A) IL-8 promoter reporter activity in MCF7-Vec and MCF7-TFF3 with depletion of STAT3 transiently transfected with an IL-8 promoter reporter vector (full length, -4800 to + 104 bp) and a pRL-CMV control reporter vector. Scrambled control siRNA was used as control. MCF7-Vec transiently transfected with scrambled control siRNA was as baseline. (B) ELISA analysis of IL-8 protein secreted to the medium by MCF7-Vec and MCF7-TFF3 transiently transfected with pcDNA vector containing STAT3 siRNA or control siRNA. *, *P < 0*.*05*; **, *P* < *0*.*01* as compared MCF7-Vec or MCF7-TFF3 transiently transfected with control siRNA. (C) HUVEC migration after 24 hours co-culture with MCF-7 cells with forced expression of TFF3 transiently transfected with control siRNA and STAT3 siRNA. (D) HUVEC invasion after 24 hours co-culture with MCF-7 cells with forced expression of TFF3 transiently transfected with control siRNA and STAT3 siRNA. (E) and (F) HUVEC tubule formation *in vitro* in the Matrigel after 12 hours co-culture with MCF-7 cells with forced expression of TFF3 transiently transfected with control siRNA and STAT3 siRNA in serum-free conditions. Total tubule length (E) and tubule number (F) was assessed. **, *P* < *0*.*01* as compared to MCF7-Vec or MCF7-TFF3 transiently transfected with control siRNA, respectively; Scrambled control siRNA was used as control; MCF7-Vec transiently transfected with scrambled control siRNA was a baseline.

TFF3 has been demonstrated to promote STAT3 activity in the mammary carcinoma cells used herein [19]. The expression of IL-8 has been demonstrated to be regulated by multiple transcription factors and particularly STAT3 [37, 38]. We therefore determined if TFF3 regulation of IL-8 is mediated by STAT3. MCF7-Vec and MCF7-TFF3 with depletion of STAT3 were transiently transfected with a full length of IL-8 promoter reporter vector (-1480 to + 104 bp) and a pRL-CMV control reporter vector. Depletion of STAT3 in MCF7-Vec and MCF7-TFF3 resulted in a small but consistently decreased IL-8 promoter activity and secretion of IL-8 protein, when compared with the respective siControl cells (Fig 7A and 7B). Hence, TFF3 regulation of IL-8 is mediated, at least partially, by STAT3.

To demonstrate the functional involvement of STAT3, HUVEC were co-cultured with MCF7-Vec and MCF7-TFF3 with or without depletion of STAT3. Depletion of STAT3 in MCF7-Vec partially decreased HUVEC migration by 36% (Fig 7C) and invasion by 31% (Fig 7D), when compared with MCF7-Vec transiently transfected with control siRNA. Depleted of STAT3 in MCF7-TFF3 decreased HUVEC migration by 47% (Fig 7C) and invasion by 58% (Fig 7D), when compared with MCF7-TFF3 transiently transfected with control siRNA. Depletion of STAT3 in MCF7-Vec partially decreased tubule formation with reduction of tubule length by 32% (Fig 7E) and tubule number by 31% (Fig 7F), when compared with MCF7-Vec transiently transfected with control siRNA. Depletion of STAT3 in MCF7-TFF3 decreased tubule length by 46% (Fig 7E) and tubule number by 58% (Fig 7F), when compared with MCF7-TFF3 transiently transfected with control siRNA. Hence, depletion of STAT3 by siRNA at least further abrogated the ability of TFF3 to stimulate angiogenic behavior of HUVEC.

### Exogenous recombinant hTFF3 directly promoted angiogenic behavior of HUVEC

**Fig 8:**
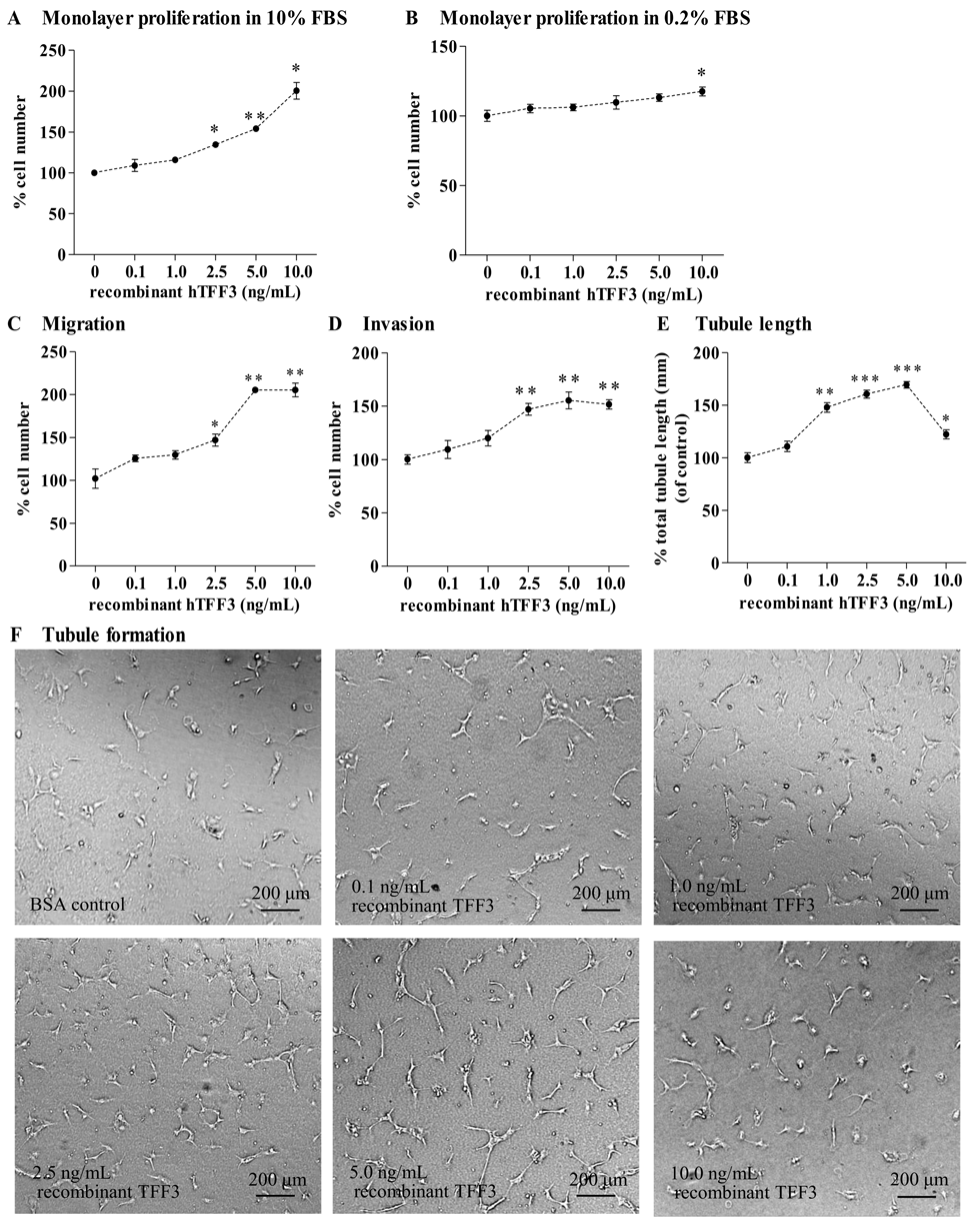
Exogenous recombinant hTFF3 increased HUVEC monolayer proliferation, migration, invasion, and tubule formation *in vitro*. (A) HUVEC monolayer proliferation at different concentrations of recombinant hTFF3 in 10% FBS condition. (B) HUVEC monolayer proliferation at different concentrations of recombinant hTFF3 in 0.2% FBS conditions. HUVEC treated with different concentrations of recombinant hTFF3 (0.1, 1.0, 2.5, 5, 10 ng/mL of recombinant hTFF3) and BSA control (10 ng/mL). BSA was used as control. HUVEC treated with BSA control was a baseline. (C) HUVEC migration after 24 hours co-cultured with different concentration of recombinant hTFF3 or BSA control. (D) HUVEC invasion after 24 hours co-cultured with different concentration of recombinant hTFF3 or BSA control. (E) HUVEC tubule formation *in vitro*, in which HUVEC treated with different concentration of recombinant hTFF3 or BSA control were plated in the Matrigel. Total tubule length was assessed using ImageJ analysis software. (F) Representative light photomicrographs of HUVEC tubule formation *in vitro*, in which HUVEC treated with different concentrations of recombinant hTFF3 or BSA control. *, *P* < *0*.*05*; **, *P* < *0*.*01*; ***, *P* < *0*.*001* as compared with BSA control. Scale bar, 200 μm.

To examine if TFF3 directly acts on HUVEC and stimulates angiogenic behavior, HUVEC were treated with different concentrations of exogenous recombinant hTFF3 (0.1, 1.0, 2.5, 5.0, and 10.0 ng/mL) and BSA control. Exogenous recombinant hTFF3 stimulation increased HUVEC monolayer proliferation from 35% at 2.5 ng/mL recombinant hTFF3 to 100% at 10.0 ng/mL recombinant hTFF3 in 10% FBS conditions when compared with HUVEC treated with BSA control protein (Fig 8A). Exogenous recombinant hTFF3 only slightly increased HUVEC monolayer proliferation in serum depleted conditions (0.2% FBS), with a small and significant increase of 18% at 10.0 ng/mL recombinant hTFF3 (Fig 8B). As compared to HUVEC co-cultured with BSA control, exogenous recombinant hTFF3 stimulation increased HUVEC migration from 45% to 104% at varying concentrations of recombinant hTFF3 (2.5, 5.0, 10.0 ng/mL of recombinant hTFF3), with the maximal HUVEC migration at 5.0 ng/mL of recombinant hTFF3 as compared with HUVEC co-cultured with BSA control (Fig 8C). Exogenous hTFF3 stimulation increased HUVEC invasion from 47% to 52% at varying concentrations of hTFF3 (2.5, 5.0, 10.0 ng/mL of recombinant hTFF3), with maximal HUVEC invasion at 5.0 ng/mL of recombinant hTFF3 as compared with HUVEC co-cultured with BSA control (Fig 8D). Exogenous hTFF3 stimulation significantly increased HUVEC tubule formation *in vitro* from 48% to 69% at varying concentration of recombinant hTFF3 (1.0, 2.5, 5.0 ng/mL of recombinant hTFF3), with maximal HUVEC tubule formation *in vitro* at 5.0 ng/mL (Fig 8E and 8F). The higher concentration of recombinant hTFF3 (10.0 ng/mL) exerted less effect on HUVEC tubule formation *in vitro* (22%) when compared with the HUVEC treated with control BSA. Hence, TFF3 also directly promotes the angiogenic behavior of endothelial cells.

## Discussion

TFF3 has previously been demonstrated to stimulate survival, invasion, and metastatic expansion of mammary carcinoma [16, 18, 19]. We [16], and numerous others, have previously reported that TFF3 is an estrogen regulated gene and is expressed predominantly in the ER+ and luminal subtype of breast cancer [18, 39, 40], and hence is also one of the genes characterizing the luminal subtype as both luminal A and luminal B are ER+. MCF-7 and T47D cells utilized herein expressed moderate levels of TFF3 and were selected as an appropriate model to study TFF3 function in ER+ mammary carcinoma cell lines. They are representative of the luminal subtype (ER+) and consistent with the pathological disease phenotype in which TFF3 is expressed. Use of these two cell lines also allows for loss of function studies on the same cell line by use of siRNA mediated depletion of TFF3. We have now demonstrated herein that mammary carcinoma derived TFF3 is a potent angiogenic factor in mammary carcinoma.

We demonstrated that TFF3 secreted by mammary carcinoma cells promoted proliferation and survival of HUVEC. Hence, TFF3 also possesses a survival function in endothelial cells. TFF3 has previously been reported to promote survival of various cancer cell lineages [16, 41, 42] and enhance resistance to serum deprivation, tamoxifen in mammary carcinoma cells [16] and drug-induced apoptosis in colon carcinoma cells [12]. One of the major survival mechanisms utilized by TFF3 is increased the expression of Bcl-2 [16], which is an anti-apoptotic protein [43] and decreased expression of Bax, which is a pro-apoptotic Bcl-2-family member [43, 44]. TFF3 function appears to be associated with multiple survival pathways including mitogen-activated protein kinase (MAPK) [45], phosphatidylinositol-3-kinase-Akt (PI3K-Akt) [41, 46], signal transducer and activator of transcription 3 (STAT3) [47] and nuclear factor kappa B (NF-κB) [42]. Enhanced Bcl2 expression is most likely a consequence of combined activation of these survival pathways, resulting from increased TFF3 expression [16]. Additionally, we have demonstrated herein that forced expression of TFF3 in mammary carcinoma cells promoted migration and invasion of HUVEC. Again, TFF3 has been reported to promote motility in a range of different cell types including Madin-Darby canine kidney cells (MDCK) [48, 49], rat fibroblastic cells [50], normal and transformed bronchial epithelial cells colonic [51] and mammary carcinoma cells [16, 19]. One of the major mechanisms by which TFF3 promotes migration and invasion appears to be repression of E-cadherin expression and/or function [12, 50, 52], although other pathways may contribute such as p44/42 MAPK [45], as p44/42 MAP kinase has been reported to decrease E-cadherin expression [52, 53].

TFF peptides have previously been suggested to possess angiogenic activities [20]. Both TFF1 and TFF3 have been reported to promote formation of capillary vessels in a CAM assay [20]. Clinically, the expression of TFF3 was positively correlated with tumor vascularity in gastric [21] and microvessel density in mammary carcinoma [18]. Herein, we have shown that TFF3 secreted from mammary carcinoma cells is a functional promoter of tumor angiogenesis as demonstrated in both *in vitro* and *in vivo* xenograft models. TFF3 exerted its angiogenic effects on endothelial cells directly as well as indirectly via enhancement of the expression of IL-8 from both mammary carcinoma and endothelial cells. IL-8 has been reported to promote angiogenic responses in endothelial cells [54] and to increase microvessel density in xenograft tumors [55, 56]. Besides promotion of tumor angiogenesis, IL-8 has been shown to increase cancer cell proliferation and invasion and promote metastasis [57, 58]. Given that these cellular processes are also stimulated by TFF3 [16, 19, 47, 48], it is reasonable to conclude that TFF3 regulated IL-8 may cooperate together with TFF3 to promote cell proliferation, invasion and metastasis of mammary carcinoma [51, 59]. For example, the expression of IL-8 is highly increased in tamoxifen resistance mammary carcinoma cells [60] as TFF3 possesses a clear functional role in tamoxifen resistant mammary carcinoma [16]. In addition to IL-8, it was noted that TFF3 modulated the expression of a number of other genes involved in angiogenesis, including TGF-β, and it is likely that the effect of TFF3 on tumor angiogenesis is the result of a co-ordinated and combinatorial pattern of gene expression. Analogously, these same set of TFF3 regulated genes may also contribute to other TFF3 regulated processes in mammary carcinoma, for example, invasion and metastasis.

Several studies have supported that the function of CXCR1 and CXCR2 in tumor angiogenesis [61, 62] and of these two receptors, CXCR2 is the receptor most likely involved in IL-8 promoted angiogenesis in endothelial cells [63, 64]. Herein, we have demonstrated that TFF3 stimulated angiogenic behavior of HUVEC are partly mediated by IL-8 through CXCR2. In contrast, CXCR1 was likely not involved in HUVEC tubule formation *in vitro* stimulated by TFF3. It is possible that the anti-CXCR2 monoclonal antibody is disrupting IL-8 independent events as CXCR2 can bind to IL-8 and multiple other angiogenic chemokines. Our observation herein is concordant with Heidemann *et al*. [65] who reported that IL-8 promoted angiogenesis in human intestinal microvascular endothelial cells is dependent on CXCR2. A number of reports have also demonstrated retardation of endothelial cell migration by a monoclonal antibody to CXCR2 [66, 67] and inhibition of vascularization in CXCR2 knockout mice [68]. We therefore conclude that TFF3 secreted from mammary carcinoma cells stimulated angiogenic behavior of HUVEC through IL-8/CXCR2 signaling.

Exogenous recombinant hTFF3 directly acted on HUVEC to promote angiogenic behavior and angiogenesis *in vitro*. It was noted that the higher concentration of exogenous recombinant hTFF3 resulted in submaximal HUVEC tubule formation *in vitro*. TFF1 and TFF3 exist in monomeric or dimeric forms with the dimeric form being multiple folds more potent [69, 70]. As with most ligands that exist in dimeric form [71], higher concentrations may result in submaximal responses with subsequent attenuation of the biological response. A similar effect of higher concentrations of TFF1 has been observed for TFF1 simulation of mammary carcinoma cell migration [70]. The functional receptor for TFF3 remains to be identified, albeit much effort has been expended to identify potential binding partners of TFF3. Numerous studies have evident that TFF3 transmits extracellular signals and promotes cell proliferation and migration through EGF-R activation [45, 72, 73] but is likely to be an indirect activation of the EGF-R to stimulate the Ras/MEK/MAPK signaling pathway [45, 74] and not that the EGF-R is the specific TFF3 receptor [75]. TFF3 has also been reported to bind to specific cell surface proteins and stimulate its functional effects independent of the EGF-R signaling pathway [49]. TFF3 binds to an unidentified 28 kDa protein expressed in MCF-7 and HT-29 cells [76] and to DMBT1 [77], IgGFcγBP and Muc2 [78], and BAT3 [79]. Nevertheless, there is no evidence indicates that any of the proteins binding TFF3 are functional TFF3 receptors and there may exist tissue specificity in these interactions. Radioligand assays have demonstrated the specific interaction between TFF3 and an unidentified protein on intestinal epithelial cells [75]. Future investigations are needed to delineate the identity of TFF3 receptor(s) that directly mediates the mechanistic action of TFF3 in angiogenesis. In any case, it is clear that endothelial cells possess a functional response to TFF3 as do mammary carcinoma cells [16, 19].

**Fig 9:**
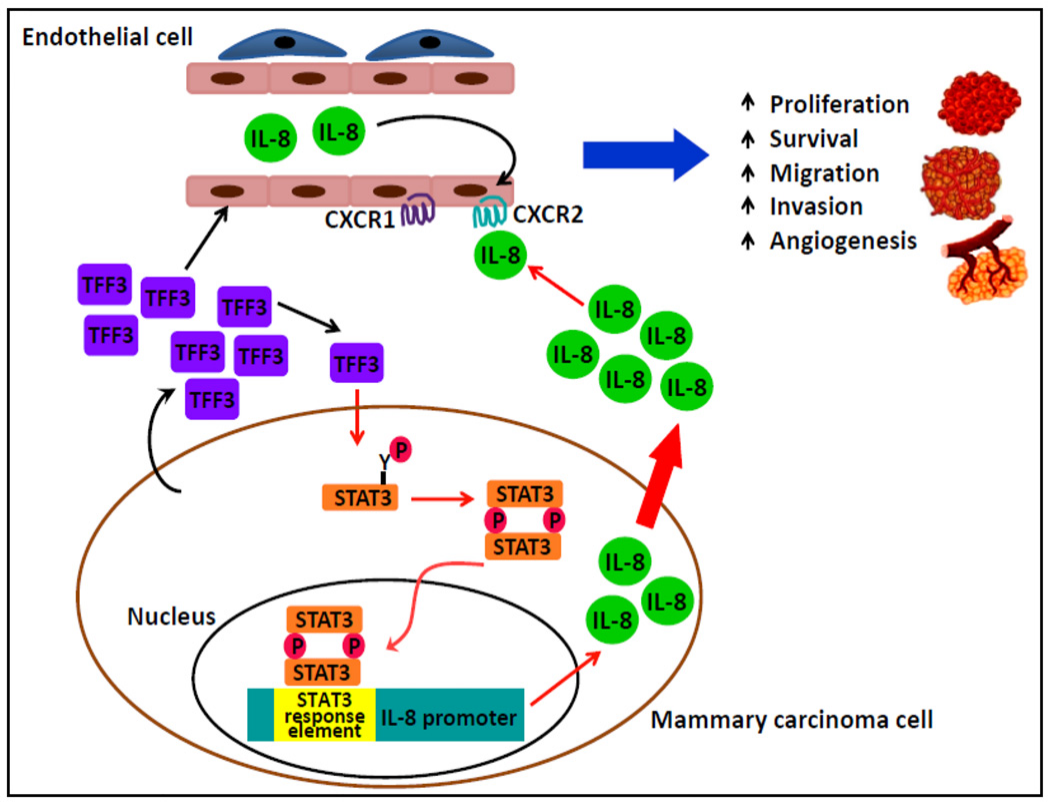
TFF3 is a promoter of angiogenesis in mammary carcinoma. TFF3 secreted from mammary carcinoma cells indirectly stimulated angiogenic behavior of endothelial cells to promote angiogenesis in mammary carcinoma via an IL-8/CXCR2 axis. STAT3 is one transcription factor responsible for the increased expression of IL-8 by TFF3. TFF3 also promotes angiogenesis by direct functional effects on endothelial cellular processes promoting angiogenesis. TFF3 stimulates angiogenesis to co-coordinate with the growth promoting and metastatic actions of TFF3 in mammary carcinoma to enhance tumor progression and dissemination.

In summary, we have demonstrated that TFF3 functions as a promoter of angiogenesis in mammary carcinoma. We have proposed a schematic model of the mechanism by which TFF3 in promotes angiogenesis in mammary carcinoma (Fig 9). TFF3 promoted *de novo* angiogenesis is mediated directly on endothelial cells and indirectly by enhanced IL-8 expression in mammary carcinoma cells. The angiogenic and metastatic actions of TFF3 therefore regulate tumor progression and dissemination of cancer cells.

## Materials and Methods

### Cell culture and transfection

The human mammary carcinoma cell lines MCF-7 and T47D were obtained from the American Type Culture Collection (Manassas, VA) and were cultured in RPMI 1640 medium supplemented with 10% heat-inactivated fetal bovine serum (FBS), 100 IU/mL penicillin, 100 μg/mL streptomycin, and 2 mM L-glutamine as recommended. Human umbilical vein endothelial cells (HUVEC) were obtained from Clonetics Solingen, Germany (a gift from Dr. Lim Yoon Pin, Department of Biochemistry, National University of Singapore). Human umbilical vein endothelial cells (HUVEC) were maintained in endothelial cell basal medium (Clonetics, Walkersville, MD) supplemented with Bullet Kit (EBM-2) on a culture flask coated with 0.1% gelatin and maintained at 37°C with humidified of 5% carbon dioxide. HUVEC cultured from passages 4 to 8 was used for the experiments. Generation of MCF-7 and T47D cells with forced expression of TFF3 or siRNA mediated depletion of TFF3 have been previously described [16].

### Preparation of total RNA and semi-quantitative RT-PCR

Total RNA was isolated from exponentially growing cells (70% confluence) using RNeasy^®^ Mini Kit (Qiagen, Limburg, Netherlands) as described by manufacturer's instruction. Semi-quantitative RT-PCR was performed by using Invitrogen^TM^ OneStep RT-PCR System with Platinum^®^ *Taq* DNA polymerase Kit (Invitrogen, Carlsbad, CA). Amplified RT-PCR products were visualized on a 2% agarose gel. Semi-quantitative RT-PCR was carried out using OneStep RT-PCR kit (Qiagen, Limburg, Netherlands). Sequences of the oligonucleotide primers for RT-PCR and specific PCR conditions were employed as previously described [80].

### Real-time qPCR analysis

The real-time quantitative PCR (qPCR) was preformed as described previously [80]. A list of angiogenic genes was analyzed by real-time qPCR and the primer sequences have been previously reported [16].

### Western blot analysis

Western blot analysis was performed as previously described [81]. The following antibodies were used: rabbit anti-TFF3 polyclonal antibody (BioGene GmBH, Berlin) and mouse anti-β-actin (Santa Cruz Biotechnology, USA). The secondary antibody anti-rabbit or anti-mouse IgG conjugated with horseradish peroxidase was from Cell Signaling Technology. Proteins were visualized using horseradish peroxidase conjugated secondary antibody with enhanced chemiluminescence ECL kit (SuperSignal West Pico substrate; Pierce, Rockford, IL).

### Quantification of secreted IL-8 protein by ELISA

IL-8 protein secreted to the conditioned medium was assayed by Human IL-8 ELISA MAX™ Deluxe kit (Biolegend, San Diego, CA) according to manufacturer's instruction. A standard curve of absorbance reading versus the different concentration of recombinant IL-8 standards was plotted. The concentration of IL-8 protein secreted to conditioned medium was determined by comparing the absorbance reading of the sample to the standard curve.

### Monolayer cell proliferation

For co-culture assay, mammary carcinoma cells with forced or depleted expression of TFF3 at density of 80,000 cells were plated on the membrane of a 0.4-μm transwell insert (BD Biosciences Pharmingen, San Diego, CA) in 10% FBS RPMI medium for 24 hours. Medium were then changed to 0.2% FBS or 10% FBS RPMI medium, respectively. Subsequently, HUVEC cells were seeded in the bottom well of six-well plates at a density of 50,000 cells per well in serum-free EGM-2 medium. The proliferation of HUVEC were counted after 24 hours incubation for consecutive two or three days. For monolayer cell proliferation assay using exogenous recombinant hTFF3, HUVEC (50,000 cells) in both 0.2% and 10% FBS EBM-2 medium containing different concentration of recombinant hTFF3 (0.1, 1.0, 2.5, 5.0 10.0 ng/mL of recombinant hTFF3) were plated in the 24-well plates in triplicate and incubated at 37°C for 48 hours. The proliferation of HUVEC were counted.

### Cell cycle progression and apoptotic cell death

Cell cycle progression was assayed by measuring the incorporation of 5-bromo-2-deoxyuridine (BrdU) into DNA of cells during the S phase of the cell cycle [82]. BrdU detection was performed as described previously [80]. A population of HUVEC cells was analyzed in multiple randomly chosen microscopic fields to determine the BrdU labeling index (percentage of cell synthesizing DNA). Apoptotic cell death was measured by fluorescent microscopic analysis of DNA staining patterns with Hoechst 33258 as previously described [82]. Apoptotic nuclear morphology was quantified with a UV fluorescence microscope (Olympus). Apoptotic cells were identified by nuclear condensation and fragmentation and including the higher intensity of blue fluorescence of the nuclei.

### Transwell migration and invasion assays

Transwell migration and invasion assays were performed using BD cell culture inserts (8.0 μm membrane pores) according to Brunet-Dunand *et al*., (2009). Transwell migration and invasion assays were conducted in which HUVEC seeded on the membrane of transwell insert and co-cultured with mammary carcinoma cells with forced or depleted expression of TFF3 plated in the bottom well of the companion plate. For migration and invasion assays using exogenous recombinant hTFF3, HUVEC (30,000 cells) in serum-free medium were seeded on the membrane of transwell insert and co-cultured with different concentration of recombinant hTFF3 added in the bottom well of the companion plate. BSA was added to control well. After 24 hours incubation, filters were rinsed with PBS, fixed with cold 4% paraformaldehyde, stained with Hoechst 33258, and the migrated cells on the underneath of whole transwell inserts were counted.

### Tubule formation assay

A total of 30,000 HUVEC were seeded in Matrigel in serum-free medium in the 24-well plates in triplicate, incubated at 37°C for 12 hours, and fixed with 4% paraformaldehyde at room temperature. Tubules were visualized by light microscopy at low magnification (×40). For co-culture assay, mammary carcinoma cells with forced or depleted expression of TFF3 were plated at 50,000 cells per transwell insert (0.4 μm membrane pores) in 10% FBS for 24 hours. Medium was changed to serum-free medium before inserts were transferred to a plate containing HUVEC seeded in Matrigel as described above. For tubule formation *in vitro* assay using exogenous recombinant hTFF3, HUVEC (30,000 cells) were treated with different concentration of recombinant hTFF3 were seeded in the Matrigel coated in the 24-well plates in triplicate. BSA was added to control wells. Photomicrographs from each well were captured, and total tubule length and number of tubule were analyzed using ImageJ software, version 2.02 (National Institutes of Health, USA).

### Transient transfection of IL-8 siRNA

The transient transfection of siRNA was performed with Lipofectamine® RNAiMAX Transfection Reagent (Invitrogen, Carlsbad, CA) according to manufacturer's instruction with slight modifications. For IL-8 gene silencing, both random sequence control siRNA and On-Target IL-8 siRNA were purchased from Dharmacon (Chicago, IL). The Smartpool On-Target IL-8 siRNA was an equal mix of four different siRNA species designed to hybridize and target human IL-8 mRNA (GenBank accession number NM_000584) for destruction. These siRNA sequences have been confirmed to be specific to IL-8 and thus avoiding potential off-target effects. Mammary carcinoma cells with forced expression of TFF3 or control vector cells were transiently transfected with 20 nM of IL-8 siRNA and scrambled control siRNA. IL-8 protein secreted to conditioned medium by mammary carcinoma cells with forced expression of TFF3 or control vector cells transiently transfected with IL-8 siRNA and control siRNA after 48 hours transfection was quantified by ELISA analysis.

### Luciferase reporter assay

Mammary carcinoma cells with forced expression of TFF3 or depleted expression of TFF3 were plated at a density of 200, 000 cells per well in 12-well plates in triplicate and cultured in RPMI medium supplemented with 10% FBS at 37°C for 24 hours before transfection. The cells were transfected with 2.0 μg of human IL-8 promoter reporter vector (-4800 to + 104 bp) (a gift from Dr. Suswam A. Esther, Department of Neurology, University of Alabama, Birmingham) and 40 ng of pRL-CMV control reporter vector (Promega, Madison, WI, USA) using FuGENE 6 reagent (Promega, Madison, WI, USA). Luciferase activity of the reporter plasmids were measured using the Dual-Luciferase Reporter Assay System (Promega, Madison, WI, USA) and with *Renilla* luciferase activity used as an internal control.

### Tumor xenograft analysis

The animals were maintained in a pathogen-free barrier facility at the Animal Center of the University of Science and Technology of China (USTC). All animal work procedures were approved by USTC Ethics Committee for Animal Care and Use and were performed in accordance with the regulations of animal care of USTC and conformed to the legal mandate and national guidelines for the care and maintenance of laboratory animals. Female BALB/c-nu/nu mice (6 weeks old) (Shanghai Slaccas Co., Shanghai, China) were used. MCF7-Vec and TFF3 cells (5 x 10^6^ cells per site) were injected s.c. into the right and left flank of female BALB/c-nu/nu mice (Shanghai Slaccas Co., Shanghai, China). Each group consisted of six nude mice. After six weeks, mice were sacrificed by CO_2_ inhalation. The tumors tissues were resected for immunohistochemistry studies. Sections were deparaffinised in xylene, rehydrated in a graded series of ethanol solutions, quenched for endogenous peroxidase activity in 3% (v/v) hydrogen peroxide, and heated in 10 mM citrate buffer (pH 6.0) at 90–100°C for 10 minutes for antigen retrieval. Sections were then incubated at 4°C for 24 hours with anti-CD31, anti-CD34, and anti-IL8 monoclonal antibodies. Antibodies used for IHC analysis including anti-IL-8 monoclonal antibody (MAB 208, R&D Systems, Minneapolis, MN), mouse anti-CD31 (550274, BD Biosciences) and mouse anti-podoplanin or anti-CD34 (ab11936, Abcam, Cambridge, UK). The sections were rinsed with PBS and incubated with biotinylated anti-rabbit IgG or anti-mouse IgG secondary antibodies added at room temperature for 1 hour. An Elite ABC immunoperoxidase kit (Vector Laboratories, CA) was used for detection.

### Densitometric analysis

The amplified PCR products were resolved by agarose gel electrophoresis and images captured by the Gel Doc imaging system (Bio-Rad, Hercules, CA). The intensity of the band of interest was analyzed by Quantity One program (version 1.0.1, Bio-Rad, Hercules, CA). The protein bands developed in X-ray film were scanned and analyzed by ImageJ Program, version 1.45s (National institute of Health, USA). Bands of interest in the agarose gel or X-ray films were identified and their total integrated volumes were quantified against the background. Density was the corrected intensity integrated with volumes (in arbitrary units).

### Statistical analysis

All experiments were repeated at least three times. All numerical data were presented as mean ± standard error of mean (SEM) and statistical significance was assessed by unpaired two-tailed Student's *t* test or analysis of variance. *P < 0*.*05* was considered as statistical significant.

## Supporting Information

S1 File Endogenous expression of TFF3 in mammary carcinoma and endothelial cells. **(**A) Semi-quantitative RT-PCR analysis of TFF3 mRNA level in MCF-7 and T47D cells. (B) Western blot analysis of TFF3 protein present in both lysate and secreted to the media of MCF-7 and T47D cells. (C) Semi-quantitative RT-PCR analysis of TFF3 mRNA level in MCF-7 cells (control) and HUVEC. (D) Western blot analysis of TFF3 protein present in lysate of MCF-7 cells (control) and HUVEC. (E) Semi-quantitative RT-PCR analysis TFF3 mRNA levels in MCF-7 cells with forced expression of TFF3 (MCF7-TFF3) and control vector cells (MCF7-Vec). (F) Western blot analysis of TFF3 protein in MCF-7 cells with forced expression of TFF3 and control vector cells. (G) Representative light photomicrographs of HUVEC cell cycle progression after 24 hours co-culture with MCF-7 cells with forced expression of TFF3 in serum-free and 10% FBS conditions. HUVEC with nuclear BrdU incorporation was stained with 3,3'-diaminobenzedine (DAB). (H) Representative fluorescent photomicrographs of HUVEC apoptotic cell death after 24 hours co-culture with MCF-7 cells with forced expression of TFF3 in serum-free and 10% FBS conditions. Apoptotic cell death of HUVEC was characterized by nuclear condensation and the higher intensity of blue fluorescence of nucleic. β-ACTIN was used as input control in semi-quantitative RT-PCR and Western blot analysis. Scale bar, 100 μm.
(TIF)

S2 File T47D cells with forced expression of TFF3 increased angiogenic behavior of HUVEC. **(**A) Semi-quantitative RT-PCR analysis of TFF3 mRNA level in T47D cells with forced expression of TFF3 (T47D-TFF3) and control vector cells (T47D-Vec). (B) Western blot analysis of TFF3 protein in T47D cells with forced expression of TFF3 and control vector cells. (C) Monolayer proliferation of HUVEC after co-culture with T47D cells with forced expression of TFF3 in 10% FBS conditions. (D) Monolayer proliferation of HUVEC after co-culture with T47D cells with forced expression of TFF3 in 0.2% FBS conditions. (E) HUVEC cell cycle progression after 24 hours co-culture with T47D cells with forced expression of TFF3 in serum-free and 10% FBS conditions. (F) HUVEC apoptotic cell death after 24 hours co-culture with T47D cells with forced expression of TFF3 in serum-free and 10% FBS conditions. (G) HUVEC migration after 24 hours co-culture with T47D cells with forced expression of TFF3 in serum-free conditions. (H) HUVEC invasion after 24 hours co-culture with T47D cells with forced expression of TFF3 in serum-free conditions. (I) and (J) HUVEC tubule formation *in vitro* in the Matrigel after 12 hours co-culture with T47D cells with forced expression of TFF3. Total tubule length (I) and total tubule number (J) were assessed. (K) Representative light photomicrographs of HUVEC tubule formation *in vitro* in Matrigel after 12 hours co-culture with T47D cells with forced expression of TFF3. T47D cells with empty vector (T47D-Vec) was used as control. β-ACTIN was used as input control in semi-quantitative RT-PCR and Western blot analyses. *, *P < 0*.*05*; **, *P* < *0*.*01*; ***, *P* < *0*.*001*; scale bar, 200 μm.
(TIF)

S3 File T47D cells with siRNA mediated depletion of TFF3 decreased angiogenic behavior of HUVEC. (A) Semi-quantitative analysis of TFF3 mRNA level in T47D cells with depletion of TFF3 (T47D-siTFF3) and control vector cells (T47D-siVec). (B) Western blot analysis of TFF3 protein in T47D cells with depletion of TFF3 and control vector cells. (C) Monolayer proliferation of HUVEC after co-culture with T47D cells with depletion of TFF3 in 10% FBS conditions. (D) Monolayer proliferation of HUVEC after co-culture with T47D cells with depletion of TFF3 in 0.2% FBS conditions. (E) HUVEC cell cycle progression after 24 hours co-culture with T47D cells with depletion of TFF3 in serum-free and 10% FBS conditions. (F) HUVEC apoptotic cell death after 24 hours co-culture with T47D cells with depletion of TFF3 in serum-free and 10% FBS conditions. (G) HUVEC migration after 24 hours co-culture with T47D cells with depletion of TFF3 in serum-free conditions. (H) HUVEC invasion after 24 hours co-culture with T47D cells with depletion of TFF3 in serum-free conditions. (I) and (J) HUVEC tubule formation *in vitro* in Matrigel after 12 hours co-culture with T47D cells with depletion of TFF3 in serum-free conditions. Total tubule length (I) and tubule number (J) were assessed after 12 hours incubation. K, representative light photomicrographs of HUVEC tubule formation *in vitro* in the Matrigel after 12 hours co-culture with T47D cells with depletion of TFF3. T47D cells with siRNA control vector (T47D-siVec) was used as control. β-ACTIN was used as input control in semi-quantitative RT-PCR and Western blot analyses. *, *P < 0*.*05*; **, *P* < *0*.*01*; ***, *P* < *0*.*001*; scale bar, 200 μm.
(TIF)

S4 File Gene expression of angiogenic markers modulated by TFF3 and IL-8 promoter activity and its expression in mammary carcinoma cells with forced expression of TFF3. (A) Real-time qPCR analysis of the gene expression of angiogenic markers modulated by TFF3. (B) IL-8 promoter reporter activity (full length, -4800 to + 104 bp) in T47D cells with forced expression of TFF3 and control vector cells. (C) Semi-quantitative RT-PCR analysis of IL-8 mRNA level in T47D cells with forced expression of TFF3 and control vector cells. (D) ELISA analysis of IL-8 protein secreted to the medium by T47D cells with forced expression of TFF3 and control vector cells. (E) IL-8 promoter reporter activity (-4800 to + 104 bp) in T47D cells with depletion of TFF3 and control vector cells. (F) Semi-quantitative RT-PCR analysis of IL-8 mRNA level in T47D cells with depletion of TFF3 and control vector cells. (G) ELISA analysis of IL-8 protein secreted to the medium by T47D cells with depletion of TFF3 and control vector cells. β-ACTIN was used as input control in both semi-quantitative RT-PCR and Western blot analyses. Fold expression indicated gene expression of angiogenic markers in MCF-7 cells with forced expression of TFF3 relative to control MCF7-Vec. Fold expression ≥ 2 indicated the gene expression of angiogenic genes was up-regulated by TFF3. *P < 0*.*05* is statistically significant. *, *P* < *0*.*05*; ***, *P* < *0*.*001*. IL-8, interleukin 8; VEGF-A, Vascular endothelial growth factor; ANGPT1, Angiopoietin 1; TNF; Tumor necrosis factor; COL18A1, Collagen alpha-1(XVIII) chain; ANGPT2, Angiopoietin 2; THBS1, Thrombospondin 1.
(TIF)

S5 File The effect of monoclonal antibody inhibition of IL-8 or CXCR1 on TFF3 stimulated HUVEC tubule formation *in vitro*. (A) HUVEC tubule formation *in vitro* in the Matrigel after 12 hours co-culture with MCF7-Vec treated with different concentrations of anti-IL-8 monoclonal antibody (2.5, 5.0, 10.0, 20.0, 50 μg/mL) or IgG control in serum-free conditions. IgG was used as control. MCF7-Vec treated with IgG control was a baseline. *, *P* < *0*.*05*; **, *P* < *0*.*01* as compared to MCF7-Vec treated with IgG control. (B) and (C), HUVEC tubule formation *in vitro*, in which MCF-7 cells with forced expression of TFF3 co-cultured with HUVEC treated with IgG control or 20 μg/mL of anti-CXCR1 monoclonal antibody. MCF7-Vec co-cultured with HUVEC treated with IgG control was as baseline. Total tubule length (B) and tubule number (C) was assessed. (D) representative light photomicrographs of HUVEC tubule formation *in vitro*, in which MCF7-Vec and MCF7-TFF3 co-cultured with HUVEC treated with IgG control or 20 μg/mL of anti-CXCR1 monoclonal antibody. ns, not significant as compared to MCF7-Vec or MCF7-TFF3 co-cultured with HUVEC treated with IgG control; scale bar, 200 μm.
(TIF)
