# Supplementary figures and images for: Trefoil Factor-3 (TFF3) Stimulates De Novo Angiogenesis in Mammary Carcinoma both Directly and Indirectly via IL-8/CXCR2
Source: PLoS One. 2015 Nov 11;10(11):e0141947. doi: 10.1371/journal.pone.0141947 (PMC4641663; doi:10.1371/journal.pone.0141947)

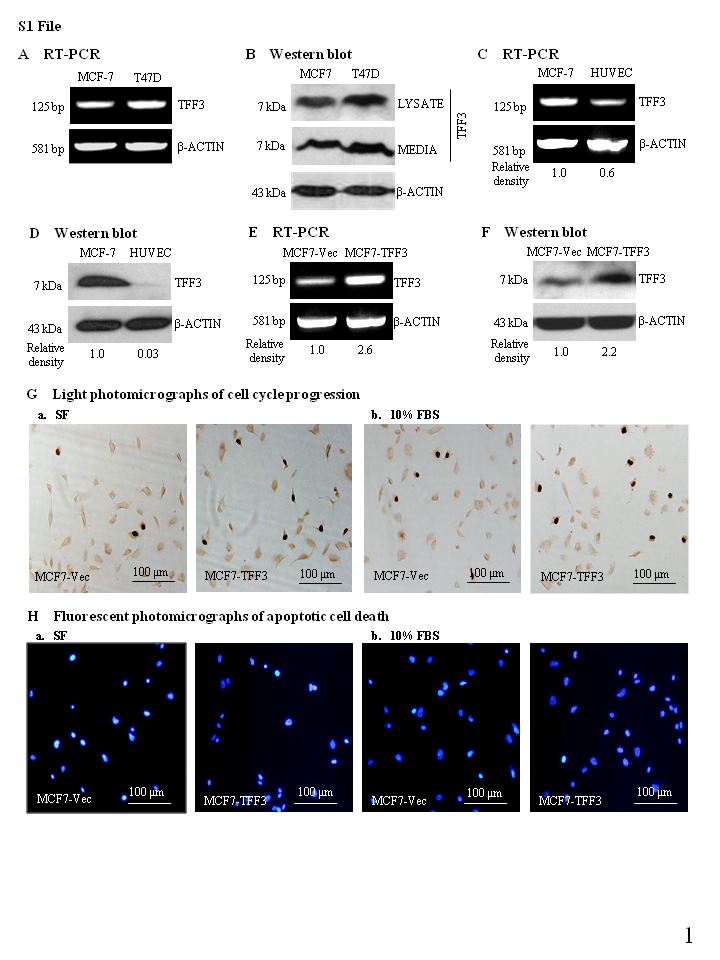

Supplement: S1 File — (A) Semi-quantitative RT-PCR analysis of TFF3 mRNA level in MCF-7 and T47D cells. (B) Western blot analysis of TFF3 protein present in both lysate and secreted to the media of MCF-7 and T47D cells. (C) Semi-quantitative RT-PCR analysis of TFF3 mRNA level in MCF-7 cells (control) and HUVEC. (D) Western blot analysis of TFF3 protein present in lysate of MCF-7 cells (control) and HUVEC. (E) Semi-quantitative RT-PCR analysis TFF3 mRNA levels in MCF-7 cells with forced expression of TFF3 (MCF7-TFF3) and control vector cells (MCF7-Vec). (F) Western blot analysis of TFF3 protein in MCF-7 cells with forced expression of TFF3 and control vector cells. (G) Representative light photomicrographs of HUVEC cell cycle progression after 24 hours co-culture with MCF-7 cells with forced expression of TFF3 in serum-free and 10% FBS conditions. HUVEC with nuclear BrdU incorporation was stained with 3,3'-diaminobenzedine (DAB). (H) Representative fluorescent photomicrographs of HUVEC apoptotic cell death after 24 hours co-culture with MCF-7 cells with forced expression of TFF3 in serum-free and 10% FBS conditions. Apoptotic cell death of HUVEC was characterized by nuclear condensation and the higher intensity of blue fluorescence of nucleic. β-ACTIN was used as input control in semi-quantitative RT-PCR and Western blot analysis. Scale bar, 100 μm. (TIF) [file pone.0141947.s001.TIF]

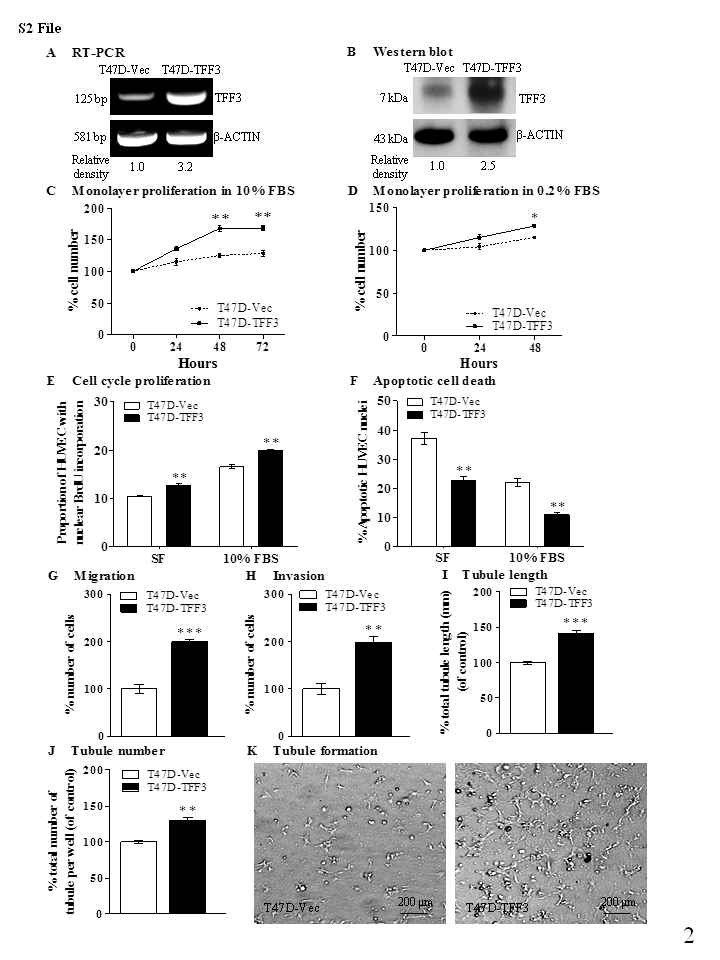

Supplement: S2 File — (A) Semi-quantitative RT-PCR analysis of TFF3 mRNA level in T47D cells with forced expression of TFF3 (T47D-TFF3) and control vector cells (T47D-Vec). (B) Western blot analysis of TFF3 protein in T47D cells with forced expression of TFF3 and control vector cells. (C) Monolayer proliferation of HUVEC after co-culture with T47D cells with forced expression of TFF3 in 10% FBS conditions. (D) Monolayer proliferation of HUVEC after co-culture with T47D cells with forced expression of TFF3 in 0.2% FBS conditions. (E) HUVEC cell cycle progression after 24 hours co-culture with T47D cells with forced expression of TFF3 in serum-free and 10% FBS conditions. (F) HUVEC apoptotic cell death after 24 hours co-culture with T47D cells with forced expression of TFF3 in serum-free and 10% FBS conditions. (G) HUVEC migration after 24 hours co-culture with T47D cells with forced expression of TFF3 in serum-free conditions. (H) HUVEC invasion after 24 hours co-culture with T47D cells with forced expression of TFF3 in serum-free conditions. (I) and (J) HUVEC tubule formation in vitro in the Matrigel after 12 hours co-culture with T47D cells with forced expression of TFF3. Total tubule length (I) and total tubule number (J) were assessed. (K) Representative light photomicrographs of HUVEC tubule formation in vitro in Matrigel after 12 hours co-culture with T47D cells with forced expression of TFF3. T47D cells with empty vector (T47D-Vec) was used as control. β-ACTIN was used as input control in semi-quantitative RT-PCR and Western blot analyses. *, P < 0.05; **, P < 0.01; ***, P < 0.001; scale bar, 200 μm. (TIF) [file pone.0141947.s002.TIF]

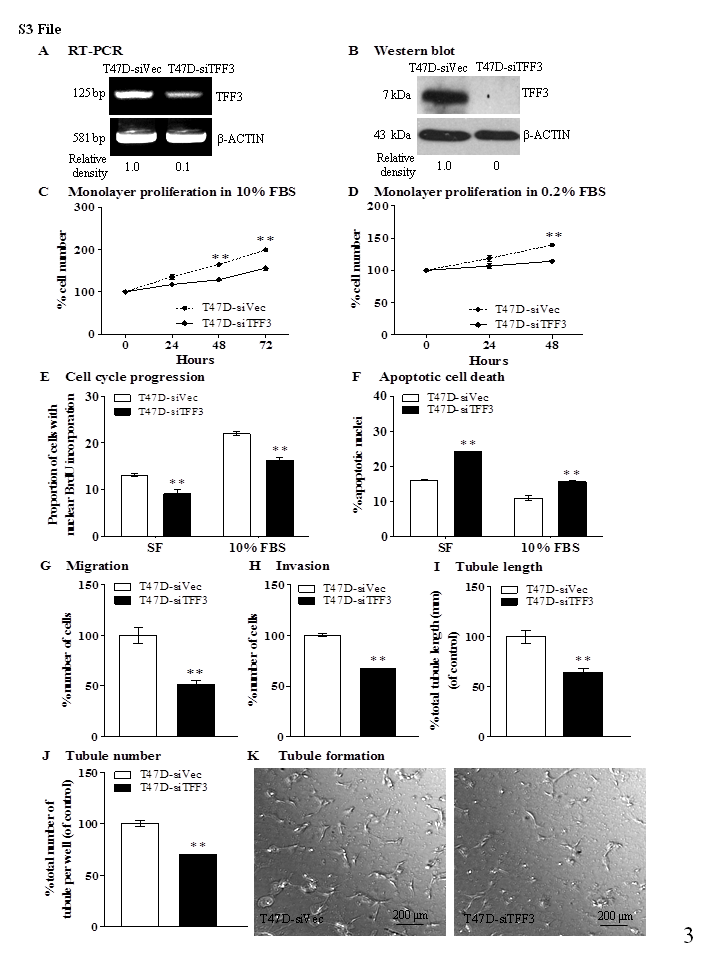

Supplement: S3 File — (A) Semi-quantitative analysis of TFF3 mRNA level in T47D cells with depletion of TFF3 (T47D-siTFF3) and control vector cells (T47D-siVec). (B) Western blot analysis of TFF3 protein in T47D cells with depletion of TFF3 and control vector cells. (C) Monolayer proliferation of HUVEC after co-culture with T47D cells with depletion of TFF3 in 10% FBS conditions. (D) Monolayer proliferation of HUVEC after co-culture with T47D cells with depletion of TFF3 in 0.2% FBS conditions. (E) HUVEC cell cycle progression after 24 hours co-culture with T47D cells with depletion of TFF3 in serum-free and 10% FBS conditions. (F) HUVEC apoptotic cell death after 24 hours co-culture with T47D cells with depletion of TFF3 in serum-free and 10% FBS conditions. (G) HUVEC migration after 24 hours co-culture with T47D cells with depletion of TFF3 in serum-free conditions. (H) HUVEC invasion after 24 hours co-culture with T47D cells with depletion of TFF3 in serum-free conditions. (I) and (J) HUVEC tubule formation in vitro in Matrigel after 12 hours co-culture with T47D cells with depletion of TFF3 in serum-free conditions. Total tubule length (I) and tubule number (J) were assessed after 12 hours incubation. K, representative light photomicrographs of HUVEC tubule formation in vitro in the Matrigel after 12 hours co-culture with T47D cells with depletion of TFF3. T47D cells with siRNA control vector (T47D-siVec) was used as control. β-ACTIN was used as input control in semi-quantitative RT-PCR and Western blot analyses. *, P < 0.05; **, P < 0.01; ***, P < 0.001; scale bar, 200 μm. (TIF) [file pone.0141947.s003.TIF]

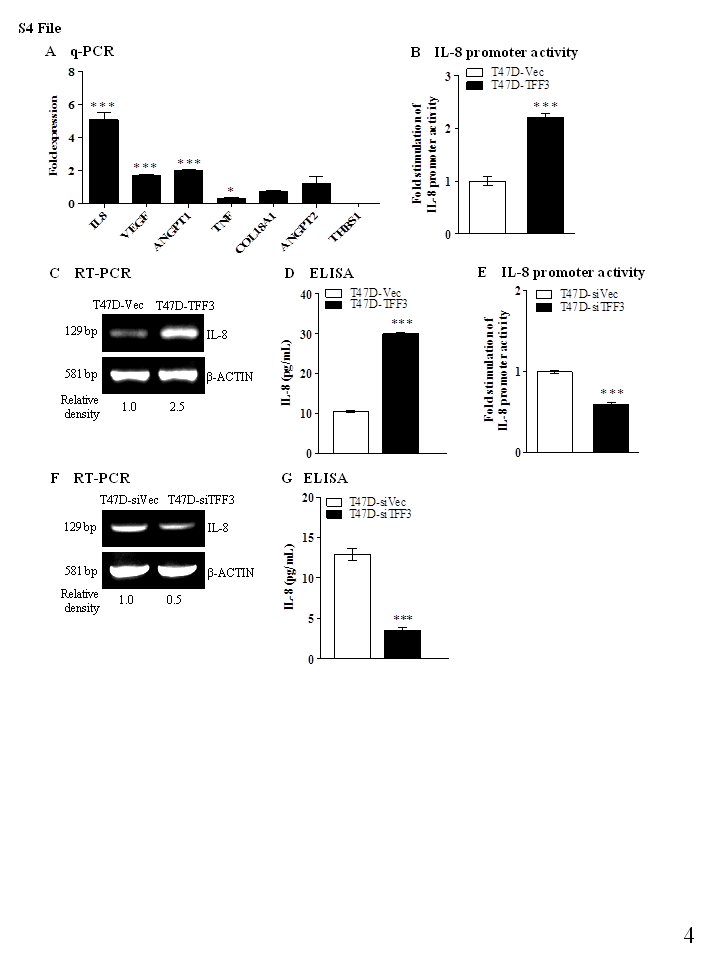

Supplement: S4 File — (A) Real-time qPCR analysis of the gene expression of angiogenic markers modulated by TFF3. (B) IL-8 promoter reporter activity (full length, -4800 to + 104 bp) in T47D cells with forced expression of TFF3 and control vector cells. (C) Semi-quantitative RT-PCR analysis of IL-8 mRNA level in T47D cells with forced expression of TFF3 and control vector cells. (D) ELISA analysis of IL-8 protein secreted to the medium by T47D cells with forced expression of TFF3 and control vector cells. (E) IL-8 promoter reporter activity (-4800 to + 104 bp) in T47D cells with depletion of TFF3 and control vector cells. (F) Semi-quantitative RT-PCR analysis of IL-8 mRNA level in T47D cells with depletion of TFF3 and control vector cells. (G) ELISA analysis of IL-8 protein secreted to the medium by T47D cells with depletion of TFF3 and control vector cells. β-ACTIN was used as input control in both semi-quantitative RT-PCR and Western blot analyses. Fold expression indicated gene expression of angiogenic markers in MCF-7 cells with forced expression of TFF3 relative to control MCF7-Vec. Fold expression ≥ 2 indicated the gene expression of angiogenic genes was up-regulated by TFF3. P < 0.05 is statistically significant. *, P < 0.05; ***, P < 0.001. IL-8, interleukin 8; VEGF-A, Vascular endothelial growth factor; ANGPT1, Angiopoietin 1; TNF; Tumor necrosis factor; COL18A1, Collagen alpha-1(XVIII) chain; ANGPT2, Angiopoietin 2; THBS1, Thrombospondin 1. (TIF) [file pone.0141947.s004.TIF]

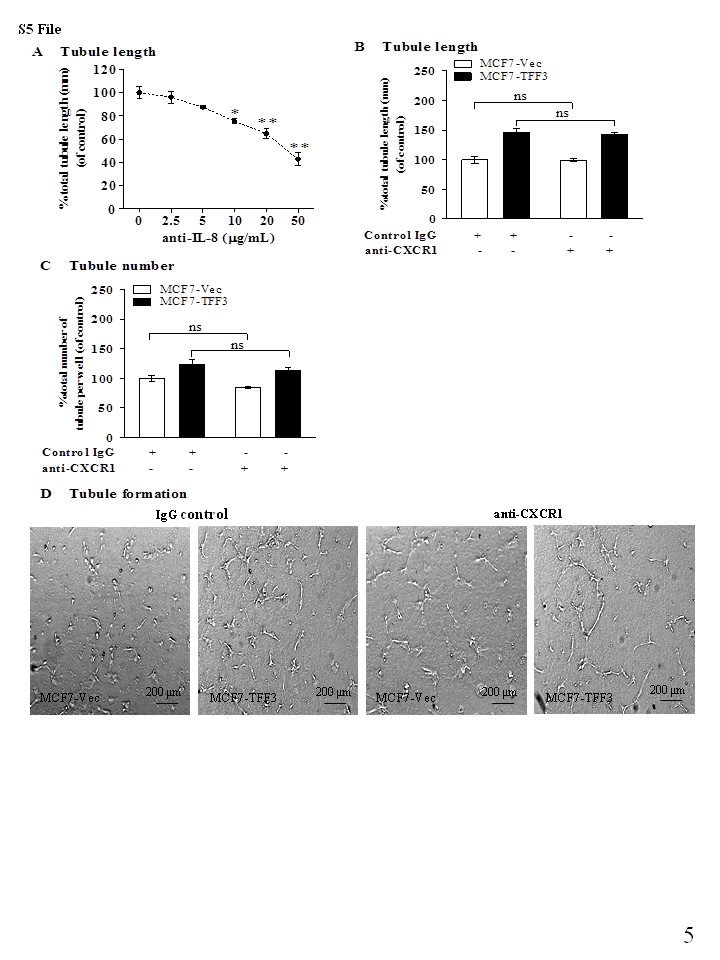

Supplement: S5 File — (A) HUVEC tubule formation in vitro in the Matrigel after 12 hours co-culture with MCF7-Vec treated with different concentrations of anti-IL-8 monoclonal antibody (2.5, 5.0, 10.0, 20.0, 50 μg/mL) or IgG control in serum-free conditions. IgG was used as control. MCF7-Vec treated with IgG control was a baseline. *, P < 0.05; **, P < 0.01 as compared to MCF7-Vec treated with IgG control. (B) and (C), HUVEC tubule formation in vitro, in which MCF-7 cells with forced expression of TFF3 co-cultured with HUVEC treated with IgG control or 20 μg/mL of anti-CXCR1 monoclonal antibody. MCF7-Vec co-cultured with HUVEC treated with IgG control was as baseline. Total tubule length (B) and tubule number (C) was assessed. (D) representative light photomicrographs of HUVEC tubule formation in vitro, in which MCF7-Vec and MCF7-TFF3 co-cultured with HUVEC treated with IgG control or 20 μg/mL of anti-CXCR1 monoclonal antibody. ns, not significant as compared to MCF7-Vec or MCF7-TFF3 co-cultured with HUVEC treated with IgG control; scale bar, 200 μm. (TIF) [file pone.0141947.s005.TIF]
